# Cell type-specific abnormalities of central nervous system in myotonic dystrophy type 1

**DOI:** 10.1093/braincomms/fcac154

**Published:** 2022-06-10

**Authors:** Masayuki Nakamori, Hiroshi Shimizu, Kotaro Ogawa, Yuhei Hasuike, Takashi Nakajima, Hidetoshi Sakurai, Toshiyuki Araki, Yukinori Okada, Akiyoshi Kakita, Hideki Mochizuki

**Affiliations:** Department of Neurology, Osaka University Graduate School of Medicine, 2-2 Yamadaoka, Suita, Osaka 565-0871, Japan; Institute for Open and Transdisciplinary Research Initiatives (OTRI), Osaka University, 1-1 Yamadaoka, Suita, Osaka 565-0871, Japan; Department of Pathology, Brain Research Institute, Niigata University, 1-757 Asahimachi, Chuo-ku, Niigata 951-8585, Japan; Department of Neurology, Osaka University Graduate School of Medicine, 2-2 Yamadaoka, Suita, Osaka 565-0871, Japan; Department of Statistical Genetics, Osaka University Graduate School of Medicine, 2-2 Yamadaoka, Suita, Osaka 565-0871, Japan; Department of Neurology, Osaka University Graduate School of Medicine, 2-2 Yamadaoka, Suita, Osaka 565-0871, Japan; Department of Neurology, National Hospital Organization Niigata National Hospital, 3-52 Akasakamachi, Kashiwazaki, Niigata 945-8585, Japan; Center for iPS Cell Research and Application (CiRA), Kyoto University, 53 Shogoin Kawahara-cho, Sakyo-ku, Kyoto 606-8507, Japan; Department of Peripheral Nervous System Research, National Institute of Neuroscience, National Center of Neurology and Psychiatry, 4-1-1 Ogawahigashimachi, Kodaira, Tokyo 187-8502, Japan; Department of Statistical Genetics, Osaka University Graduate School of Medicine, 2-2 Yamadaoka, Suita, Osaka 565-0871, Japan; Department of Pathology, Brain Research Institute, Niigata University, 1-757 Asahimachi, Chuo-ku, Niigata 951-8585, Japan; Department of Neurology, Osaka University Graduate School of Medicine, 2-2 Yamadaoka, Suita, Osaka 565-0871, Japan

**Keywords:** CAMKK2, CpG methylation, laser-capture microdissection, myotonic dystrophy, repeat expansion

## Abstract

Myotonic dystrophy type 1 is a multisystem genetic disorder involving the muscle, heart and CNS. It is caused by toxic RNA transcription from expanded CTG repeats in the 3′-untranslated region of *DMPK*, leading to dysregulated splicing of various genes and multisystemic symptoms. Although aberrant splicing of several genes has been identified as the cause of some muscular symptoms, the pathogenesis of CNS symptoms prevalent in patients with myotonic dystrophy type 1 remains unelucidated, possibly due to a limitation in studying a diverse mixture of different cell types, including neuronal cells and glial cells. Previous studies revealed neuronal loss in the cortex, myelin loss in the white matter and the presence of axonal neuropathy in patients with myotonic dystrophy type 1. To elucidate the CNS pathogenesis, we investigated cell type-specific abnormalities in cortical neurons, white matter glial cells and spinal motor neurons via laser-capture microdissection. We observed that the CTG repeat instability and cytosine–phosphate–guanine (CpG) methylation status varied among the CNS cell lineages; cortical neurons had more unstable and longer repeats with higher CpG methylation than white matter glial cells, and spinal motor neurons had more stable repeats with lower methylation status. We also identified splicing abnormalities in each CNS cell lineage, such as *DLGAP1* in white matter glial cells and *CAMKK2* in spinal motor neurons. Furthermore, we demonstrated that aberrant splicing of *CAMKK2* is associated with abnormal neurite morphology in myotonic dystrophy type 1 motor neurons. Our laser-capture microdissection-based study revealed cell type-dependent genetic, epigenetic and splicing abnormalities in myotonic dystrophy type 1 CNS, indicating the significant potential of cell type-specific analysis in elucidating the CNS pathogenesis.

## Teaser

Central nervous system symptoms in patients with myotonic dystrophy significantly interfere with the quality of life. Nakamori *et al.* report cell type-specific genetic, epigenetic and transcriptomic characteristics in cortical neurons, white matter glial cells and spinal motor neurons of the patients, providing new insights into the pathogenesis of myotonic dystrophy.

## Graphical Abstract

**Graphical Abstract:**
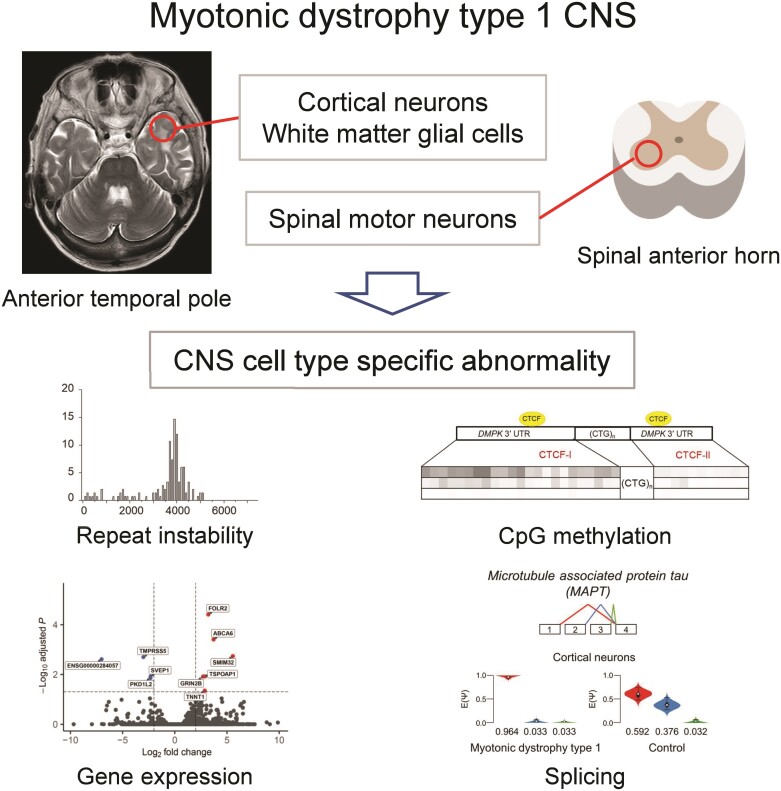

## Introduction

Myotonic dystrophy type 1 (DM1) is a prevalent type of muscular dystrophy estimated to affect 1:2100 persons.^1^ This systemic disease affects multiple organs, such as skeletal and cardiac muscles, CNS and eyes. Consequently, it presents with multisystemic symptoms including myotonia, progressive muscle wasting, insulin resistance, cardiac conduction defects, cognitive dysfunction and cataract.^2^ In many patients with DM1, CNS symptoms, such as cognitive and concentration disturbances, reduced attention and flexibility of thinking, avoidant behavioural trait, and excessive daytime sleepiness, jeopardize the ability to work and reduce the quality of life more than muscular symptoms.^3,4^ This also underlines the importance of considering CNS involvement biomarkers for therapeutic trials.^5^

DM1 is caused by a genetic defect involving the expansion of a CTG repeat in the 3′ untranslated region (UTR) of *DMPK* at 19q13.3, resulting in transcription of toxic RNAs that contain an expanded CUG repeat.^2^ In turn, toxic RNAs alter the activity of RNA binding proteins involved in alternative splicing, such as muscleblind-like splicing regulator (MBNL) and CUGBP Elav-like family member 1 (CELF1), eventually perturbing the regulation of pre-mRNA splicing in affected tissues. Thus far, numerous misregulated splicing events (>100) have been reported in DM1.^6–8^ For instance, myotonia and insulin resistance are attributed to aberrant alternative splicing of the muscle-specific chloride channel (*CLCN1*) and insulin receptor (*INSR*), respectively.^9–11^ Splicing misregulation of the voltage gated sodium channel (*SCN5A*) in the DM1 heart contributes to cardiac conduction defects and arrhythmia.^7^ Furthermore, progressive muscle wasting in DM1 muscle has been suggested to be caused due to aberrant splicing of *BIN1*, the voltage gated calcium channel (*CACNA1S*) and dystrophin (*DMD*).^12–15^ However, although previous studies using bulk brain samples revealed many misregulated splicing events in DM1 brain,^8,16–19^ the pathogenesis of CNS symptoms remains unclear. A major hindrance to elucidating the pathomechanism is that the CNS, unlike skeletal and cardiac muscles, contains a diverse mixture of different types of cells, including neuronal and glial cells. Moreover, the properties of these CNS cells vary from site to site in the brain.^20^ Neuropathological and neuroradiological studies on DM1 pointed out differences in abnormalities in the cerebral cortex and white matter.^21,22^ Furthermore, this is compounded by differences in expanded CTG repeat length, cytosine–phosphate–guanine (CpG) methylation status around the repeat tract and splicing abnormalities among cell types in patients with DM1.^6,8,23–25^ Therefore, it is essential to analyze each CNS cell type in a specific region individually to elucidate the CNS pathogenesis in DM1. The most effective method to discriminate cell type lineage in clinical samples is laser-capture microdissection (LCM).^26^ This technique provides pure cell type populations of targeted cells from specific microscopic regions of tissue sections. We employed the LCM-based approach to investigate cell type-specific abnormalities in the DM1 CNS. We comprehensively determined the CTG repeat size, CpG methylation, alternative splicing and gene expression in neuronal cells in the cortex, glial cells in the white matter and spinal motor neurons. Furthermore, we studied the relevance of disease-associated splicing abnormality in human-induced pluripotent stem cells (hiPSCs)-derived motor neurons from patients with DM1.

## Materials and methods

### Study approval

The institutional ethics committees of Osaka University and Niigata University approved this study. The study subjects provided informed consent.

### Histology and immunohistochemistry

Histological analysis was performed on 4 μm-thick sections cut from formalin-fixed, paraffin-embedded CNS tissue blocks, via haematoxylin–eosin (HE) and Klüver–Barrera (KB) staining and Gallyas–Braak silver impregnation. Immunohistochemical analyses were performed as described previously,^27,28^ using mouse monoclonal antibodies against hyperphosphorylated tau (AT8; Invitrogen, AB_223647; 1:200), phosphorylated neurofilament H (SMI31; BioLegend, AB_2564641; 1:2000), amyloid-β (12B2; IBL, 10027; 1:50), phosphorylated α-synuclein (pSyn#64; Fujifilm Wako, 015-25191; 1:1000) and ubiquitin (1B3; MBL, MK-12-3; 1:1000), as well as using a rabbit polyclonal antibody against glial fibrillary acidic protein (GFAP) (Invitrogen, AB_10980769; 1:400). The distribution of neurofibrillary tangles (NFTs) was evaluated according to Braak *et al.*^29^ Frozen samples of necropsied muscle tissue were routinely processed and stained with HE and Gomori trichrome.

### Laser-capture microdissection of CNS samples

Human brain (temporal lobe) tissues and spinal cords were obtained from five patients with DM1 and three cases of disease control (Table 1). All five patients with DM1 showed phosphorylated tau-positive NFTs and neurites in the medial temporal lobe, with distribution patterns corresponding to Braak stage I (DM1-Patient 3), Braak stage II (DM1-Patients 1, 2 and 5) and Braak stage III (DM1-Patient 4) (Table 1 and Supplementary Fig. 1). In DM1-Patient 1, phosphorylated α-synuclein-positive Lewy bodies and neurites were sparse but relatively widespread in the CNS, including the cerebral neocortex, limbic lobe and brainstem nuclei such as the dorsal nucleus of the vagal nerve (Supplementary Table 1). This distribution pattern appeared compatible with Lewy pathology in DM1.^30^ The above phosphorylated tau- or α-synuclein-positive structures were partly positive for ubiquitin. GFAP immunohistochemistry disclosed mild gliosis in the white matter of the temporal pole in DM1-Patients 1, 2 and 3 (Supplementary Table 1).

**Table 1:** Clinicopathological features of patients with DM1 and disease controls

Patient	Gender	Age at autopsy	Age at onset (category)	ePAL	Temporal lobe (cortical neurons and white matter glial cells)	Spinal cord (motor neurons)	Cognitive dysfunction	White matter hyperintense legions (WMHI)	White matter degeneration with myelin and axonal loss	Braak stage (neurofibrillary tangles)
DM1-Patient 1 (DM1-Pt 1)	F	61	30 (adult)	174	✓	✓	N/A	+	+	II
DM1-Patient 2 (DM1-Pt 2)	F	53	20 (adult)	108	✓	N/A	✓	+	+	II
DM1-Patient 3 (DM1-Pt 3)	M	48	13 (juvenile)	110	✓	N/A	✓	+	+	I
DM1-Patient 4 (DM1-Pt 4)	F	65	53 (adult)	381	N/A	✓	✓	N/A	N/A	III
DM1-Patient 5 (DM1-Pt 5)	F	47	38 (adult)	577	N/A	✓	✓	N/A	N/A	II
Control-1 (aortic dissection)	M	89			✓	✓		−	−	II
Control-2 (pontine infarction)	M	85			✓	✓		−	−	I
Control-3 (subarachnoid haemorrhage)	M	39			✓	✓		−	−	0

N/A, not available.

The frozen CNS tissues embedded in O.C.T. compound (Tissue-Tek), were sectioned (10 μm) and then mounted on membrane-coated 1 mm PEN slides (Carl Zeiss). The PALM MicroBeam System (Carl Zeiss) was used to perform LCM per the manufacturer's instructions. HE section was used as a counterstain to confirm cell specificity. Catapulted cells (at least 2000 nuclei from each brain sample and 700 nuclei from each spinal cord sample) were transferred into an RNase-free microtube for extraction and purification of nucleic acids (DNA and RNA) using an ALLPrep DNA/RNA Micro Kit (Qiagen).

### Analysis of CTG repeat size in CNS tissues of patients with myotonic dystrophy type 1

Small-pool PCR was used to size expanded CTG repeats followed by Southern blot detection, as previously described.^6^ Briefly, amplification of diluted genomic DNA was performed using an Expand Long Template PCR System (Roche) with primers 5′-ACCCTAGAACTGTCTTCGACTCC-3′ and 5′-TTCCCGAGTAAGCAGGCAGAG-3′ through a total of 24 cycles. Detection of amplicons was done through Southern blot using a digoxigenin-labeled (CAG)_7_ locked nucleic acid probe.^31^ At least 150 alleles were analyzed in each sample. The lower boundary of the expanded molecules in small-pool PCR was used to estimate the progenitor allele length (ePAL), as described previously.^32^

### Analysis of cytosine–phosphate–guanine methylation

The CpG methylation status was studied, as previously described.^33^ Briefly, bisulphite modification was performed using EpiTect Bisulphite Kit (Qiagen). PCR was used to amplify upstream and downstream regions of CTG repeats in the *DMPK* locus of purified DNA. This was followed by AMPure XP (Beckman Coulter Life Sciences) amplicons purification and subsequent DNA library preparation using the Ion Fragment Library and Ion Xpress Barcode Adaptors Kits (Thermo Fisher Scientific). Emulsion PCR was used for library amplification using Ion OneTouch 2 and Ion PGM template OT2 400 Kits (Thermo Fisher Scientific). The Ion OneTouch ES System (Thermo Fisher Scientific) was used to purify the emulsion PCR product, which was loaded onto an Ion 318 Chip (Thermo Fisher Scientific). Ion PGM sequencer and Ion PGM sequencing 400 Kit (Thermo Fisher Scientific) were used for sequencing. Resultant sequences were converted to CpG methylation statuses using Bismark (Babraham Bioinformatics).

### RNA-seq analysis

The SMART-Seq V4 Ultra Low Input RNA (Clontech) and Nextera Library Preparation (Illumina) kits were used to prepare RNA-seq libraries, which were then sequenced using an Illumina HiSeq 2500, as previously described.^34^

For gene expression analysis, the quality of sequence read data was verified using FastQC version 0.11.8. Adapter and quality trimming of sequence reads was performed using Trimmomatic version 0.36,^35^ followed by alignment with human reference genome (GRCh38) sequence using STAR version 2.5.3a software.^36^ Resultant bam files were processed using RSEM version 1.3.3^37^ and normalized using DESeq2 version 1.33.1.^38^ Principal component analysis was performed by pcaExplorer using the top 500 most significantly differentially expressed genes.^39^ Differentially expressed genes were defined by using Benjamin-Hochberg false discovery rate approach (adjusted *P* < 0.05, the absolute value of log_2_-fold change > 2.0). Gene ontology (GO) enrichment analyses were performed on differentially expressed gene sets with *P* < 0.01 and |log_2_-fold change| > 2.0 using goseq v2.12.0^40^ and Metascape (http://www.metascape.org).

For detecting alternative splicing events, Modeling Alternative Junction Inclusion Quantification (MAJIQ), version 2.1-c3da3ce^41^ was used to identify and quantify local splice variation (LSVs) from the aligned reads annotated using GENCODE (release 29). The results of alternative splicing analysis were visualized using Voila, version 2.2.0.^41^ Reverse transcriptase (RT)-PCR analysis of alternative splicing was performed using gene specific primers (Supplementary Table 2), as previously described.^24^ Quantitative RT-PCR was performed using TaqMan Gene Expression assays on an ABI PRISM 7900HT Sequence Detection System (Applied Biosystems). The level of target mRNA was determined using the delta-delta Ct method with *18S* rRNA as an endogenous control.^42^

### Human iPSC-derived motor neuron differentiation

Four hiPSC lines, HPS1051 (DM1), HPS1052 (DM1), HPS2478 (normal control) and HPS2496 (normal control) were obtained from RIKEN BRC Cell Bank. Motor neuron differentiation was performed in accordance with a previously described procedure,^43^ although with slight modifications. Briefly, hiPSCs were dissociated with 0.5 × TrypLE Select (Thermo Fisher Scientific) and then plated on Matrigel-coated plates (8.5 × 10^5 ^cells/well). On the same day, StemFit AK02N medium (Ajinomoto) was replaced with neural medium, consisting of: DMEM/F12, Neurobasal medium at 1:1, 0.5 × N2, 0.5 × B27 (Thermo Fisher Scientific), 0.1 mM ascorbic acid (Sigma), 0.5 × Glutamax and 1 × penicillin/streptomycin (Thermo Fisher Scientific). The media was supplemented with CHIR99021 (3 µM, Fujifilm Wako), 2 µM DMH1 (Sigma), 2 µM SB431542 (Fujifilm Wako) and 10 µM Y-27632 (Fujifilm Wako). The culture medium was changed every other day. Human iPSCs maintained under these conditions for 6 days were induced into neuroepithelial progenitors (NEP) cells. NEP cells dissociation was carried out using 0.5 × TrypLE Select and split at 1:4 with the neural medium. Further media additives included Retinoic acid (RA, 0.1 µM, Sigma), 0.5 µM Purmorphamine (Pur, Merck), 1 µM CHIR99021, 2 µM DMH1 and 2 µM SB431542. The medium change occurred every other day. NEP cells were maintained under these conditions for 6 days differentiated into motor neuron progenitors (MNPs). To induce motor neuron (MN) differentiation, the MNPs were dissociated with Dispase (1 mg/ml) and then cultured in suspension in a neural medium supplemented with 0.1 µM RA and 0.5 µM Pur for 6 days with medium change occurring every other day. Differentiated MNs were then plated on poly-L-ornithine/laminin-coated plates. This was followed by MN cell culture in neural medium with 0.5 µM RA, 0.1 µM Pur and 0.1 µM Compound E (Sigma) for 8 days to mature. For RNA analysis, total mRNA was extracted from differentiated MNs by using an RNeasy Micro Kit (Qiagen). First-strand complementary DNA (cDNA) was synthesized as described previously.^24^ Immunofluorescent analysis of differentiated MNs was performed as follows: cell fixing was done using 4% PFA in PBS for 15 min; standard immunofluorescence staining procedures were performed using an anti-beta-III tubulin (TUJ1) antibody (1:500; Ab18207, Abcam) visualized with a donkey anti-rabbit secondary antibody Alexa 488 (1:1000; A21206, Thermo Fisher Scientific) and counterstained with Hoechst bisbenzimide 33342 (Thermo Fisher Scientific). Imaging was performed using a BZ-X710 fluorescent microscope (Keyence).

### Axon branching counting

Axon branching in differentiated MNs was counted, as previously described with slight modification.^44^ Briefly, neurites that were >1 mm away from the somato dendrites were selected as axons. Branching numbers were counted along 250 µm of the axon terminal (within 300 µm of the distal end of axons, excluding 50 µm of the axon end). Axon branching was defined as a branching length of >2 µm. Branches from the sole axon were counted, whereas spines or axon branching originating from other branches were excluded. Neurite length was measured from the neurite end to the sphere edge using the FIJI-ImageJ, Simple Neurite Tracer plugin.^45^ To reduce bias, counting of structures and lengths was carried out in a blinded manner after random shuffling of acquired images. Ten axons were analyzed in each experiment.

### Statistical analysis

For CTG repeat size analyses, the Mann–Whitney U-test with Bonferroni’s correction was used to compare the intra-patient differences. The coefficient of variation (CV) was calculated as the ratio between the standard deviation and the mean of CTG repeat size. Differences in axon branching and gene expression were assessed by a two-tailed Student's *t*-test and considered statistically significant with *P* < 0.05. Comparisons of the average number of axon branching and axon length in each MNs line were analyzed by two-way ANOVA with *post hoc* Tukey's honestly significant difference (HSD) tests.

## Results

### Laser-capture microdissection of CNS cells

**Figure 1:**
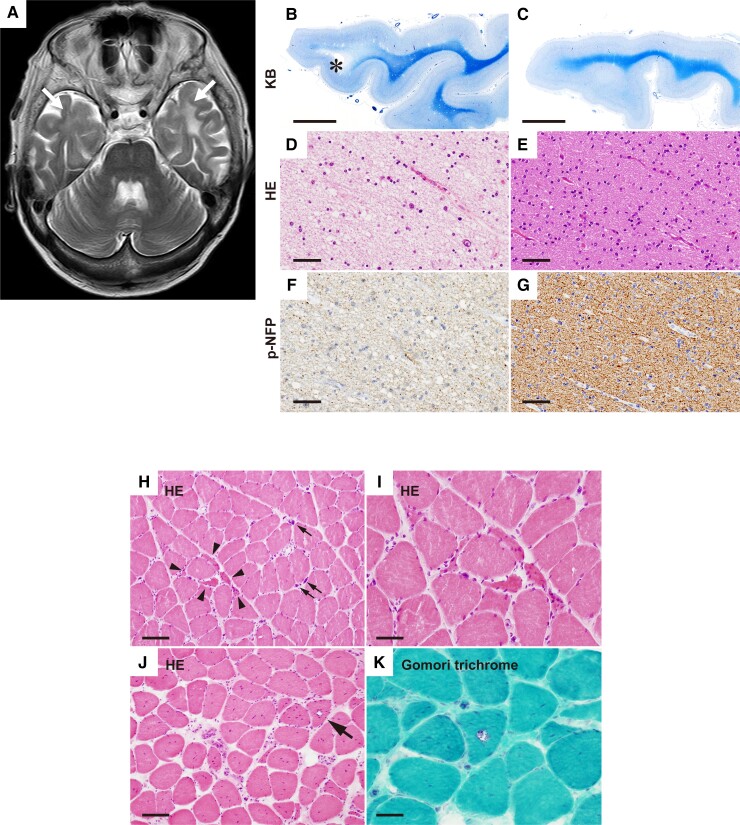
**Brain MRI image and histopathology of patients with DM1.** (**A**) Brain MRI T_2_-weighted axial image showing bilateral WMHI (white arrows) in the anterior temporal pole of DM1-Patient 2. (**B–G**) Histopathology of the temporal lobe (KB staining), (**B** and **C**); HE staining, (**D** and **E**); phosphorylated neurofilament protein (p-NFP) staining, (**F,G**). Compared with a normal control subject (**C, E, G**), DM1-Patient 2 showed marked white matter degeneration with focal cystic change (**B,** asterisk), characterized by tissue rarefaction (**D**) and loss of both myelin (**B**) and p-NFP-positive axons (**F**). Bars indicate 5 mm for (**B** and **C**) and 50 μm for (**D–G)**. (**H–K**) Histopathology of the autopsied skeletal muscles of DM1-Patient 5. HE staining of the biceps brachii shows mild variation in muscle fibre size and formation of pyknotic nuclear clumps (**H,** arrows). Clusters of small angulated fibres are also evident (**H,** arrowheads; **I**). The sternocleidomastoid muscle shows marked variation in muscle fibre size and endomysial fibrosis (**J** and **K**). Muscle fibre nuclei are increased in number and internalized. A small number of rimmed vacuoles are also evident (**J**, arrow; **K**). Bars indicate 100 μm for (**H** and **J)** and 50 μm for (**I** and **K**).

Histological studies showed the CNS pathology of DM1 is characterized by myelin loss and gliosis in the deep white matter and neuronal loss in the cortex.^21^ Neuroimaging studies in DM1 have shown white matter hyperintense lesions (WMHI) in the frontal and temporal lobe, especially in the anterior temporal pole, and widespread reductions of grey matter volume in all the cortical lobes.^22^ Based on these findings from neuropathological and neuroimaging studies, we performed LCM in the anterior temporal pole (in the temporal lobe) from three patients with DM1 showing the characteristic WMHI and pathological changes (see Materials and methods) and from three disease controls (Fig. 1A–G, Supplementary Figs. 1 and 2, Table 1 and Supplementary Table 1). We collected nuclei of neuronal cells in the cortex and glial cells in the white matter for subsequent molecular analyses (Supplementary Fig. 3).

Several studies also suggest the involvement of spinal motor neurons in the pathogenesis of DM1. For example, ribonuclear aggregation of toxic RNA was observed in the nucleus of spinal motor neurons in patients with DM1.^46^ In DM1 skeletal muscle, histological changes of denervation, such as angular atrophic fibres and pyknotic nuclear clumps, often occur;^47^ in our case, they were in DM1-Patient 5 (Fig. 1H–K). Moreover, many neurophysiological studies have suggested the presence of axonal neuropathy in patients with DM1.^48–50^ Furthermore, to investigate the pathogenesis of DM1 in spinal motor neurons, we performed LCM in spinal anterior horns and collected the nuclei from motor neurons (Supplementary Fig. 3).

### Cell type-specific variability of expanded CTG repeat size in CNS of patients with myotonic dystrophy type 1

**Figure 2:**
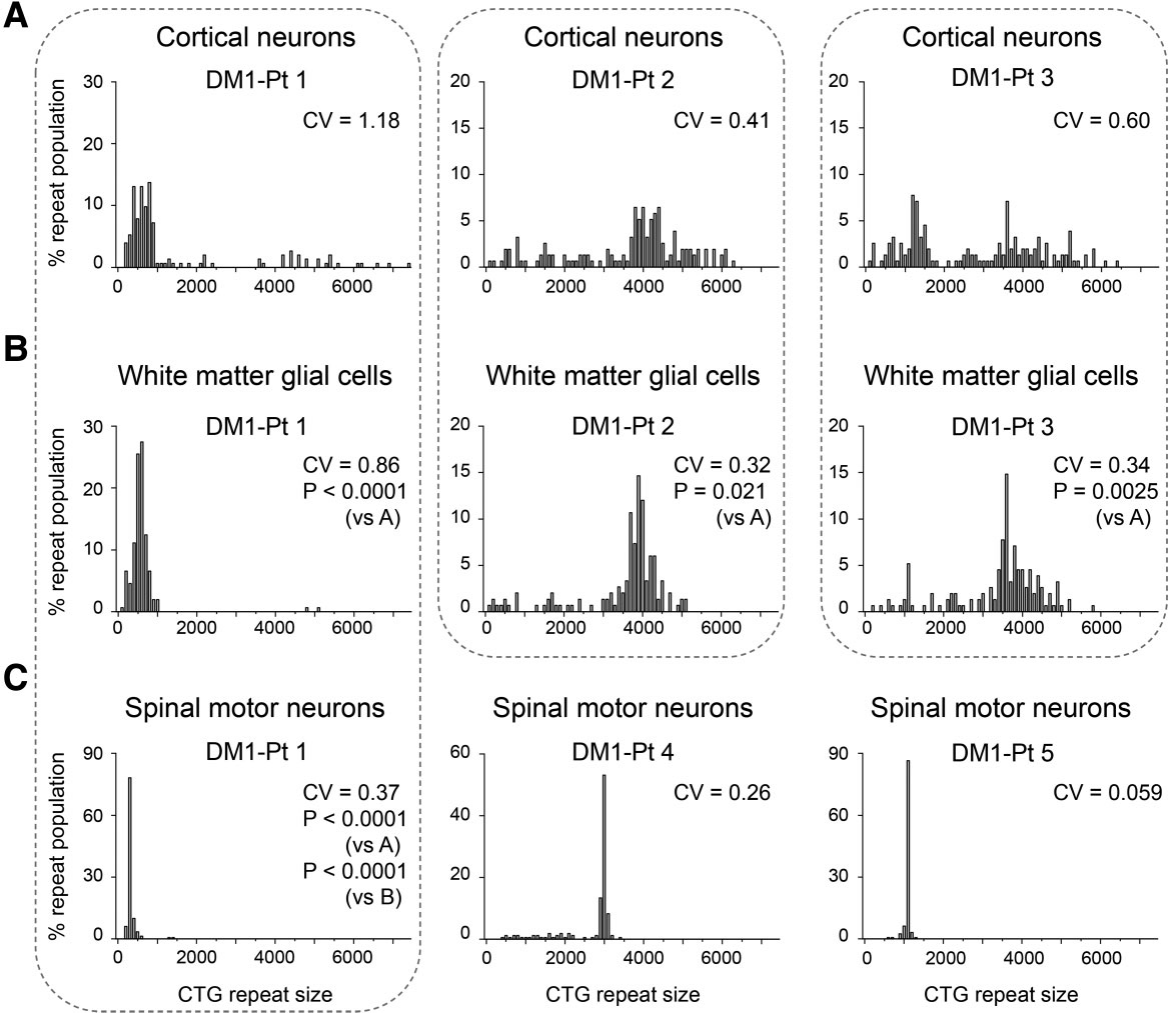
**Cell type-specific somatic repeat instability in the CNS of patients with DM1**. (**A–C**) Histograms of CTG expansions allelic distributions in cortical neurons (**A**), white matter glial cell (**B**) and spinal motor neurons (**C**) from patients with DM1. CTG expansions are binned in groups spanning 100 repeats. Dotted rectangles indicate a comparison of repeat instability and length in the same patient. CV: coefficient of variation. P: *P*-values calculated by the Mann–Whitney U-test. Because of the multiple comparisons in DM1-Patient 1 (DM1-Pt 1), the appropriate significance level was determined by Bonferroni correction, requiring a *P* < 0.0167 to be significant at the 95% level.

The length of expanded CTG repeats in DM1 varies considerably among tissues, tending to be longer in the skeletal and cardiac muscle, whereas it is shorter in the cerebellum.^25,31^ Although the repeat length in DM1 cerebral cortex is relatively longer than the other tissues,^25^ the repeat length and degree of repeat instability in each CNS cell lineage are unknown. To address this issue, we analyzed the repeat length and somatic repeat instability by small-pool PCR in cortical neurons and white matter glial cells collected from the anterior temporal pole. In all three patients with DM1, cortical neurons tended to have more markedly expanded repeats and greater repeat instability than white matter glial cells (Fig. 2A and B and Supplementary Fig. 4). We also evaluated repeat length and instability in DM1 spinal motor neurons, which had never been assessed before. In the spinal motor neurons, the repeat was much more stable and the length was shorter than those in cortical neurons and white matter glial cells in the same patient (DM1-Patient 1) (Fig. 2A–C and Supplementary Fig. 4). In spinal motor neurons derived from two other patients with DM1 (DM1-Patients 4 and 5), the repeat length was also found to be stable (Fig. 2C). These results indicate that in DM1 CNS cells, cortical neurons had unstable and longer repeats than white matter glial cells, and spinal motor neurons had shorter repeats with less instability.

### Cell type-specific variability of cytosine–phosphate–guanine methylation in CNS of patients with myotonic dystrophy type 1

**Figure 3:**
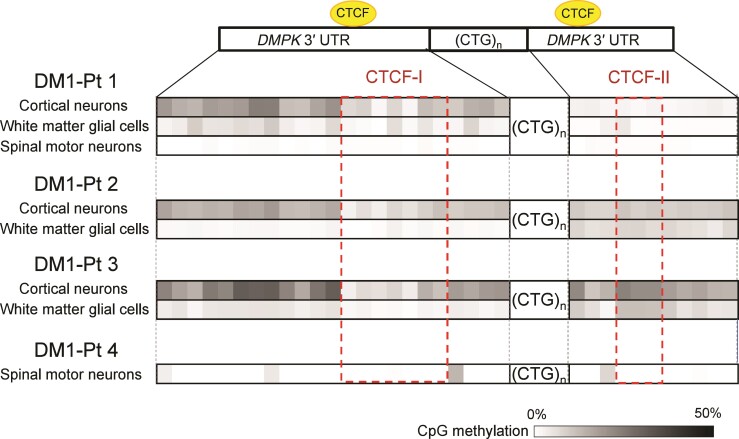
**Cell type-specific CpG methylation profiles around CTG repeats in the CNS of patients with DM1**. Heatmap of methylation levels (black, 50% methylation; white, 0% methylation) at CpG sites in the *DMPK* 3′ UTR. Dotted red boxes indicate CTCF binding sites (CTCF-I and CTCF-II).

Highly expanded CTG repeats in DM1 are suggested to associate with aberrant CpG methylation around the repeat tract.^51^ In particular, CpG methylation status in a binding site for the insulator protein CTCF upstream of the CTG repeats (CTCF-I) correlates with repeat length in skeletal muscles of congenital patients with DM1 [congenital DM1 (CDM)].^33^ The aberrant CpG methylation around the expanded repeats in DM1 varies significantly from tissue to tissue, even in the same patient.^25^ In our study, to investigate cell type-specific CpG methylation abnormalities in the CNS cell lineages with different repeat instability, we performed next-generation sequencing following bisulphite modification and quantified the methylation status of each CpG site around the repeat tract. Similar to previous studies in adult DM1 tissues and CDM skeletal muscles,^25,33^ DM1 brain tissues showed increased CpG methylation status around the CTG repeats with substantial variation among the CNS cell lineages (Fig. 3). In all three DM1 cases we analyzed (DM1-Patients 1–3), the CpG sites including the CTCF-I were more highly methylated in cortical neurons than white matter glial cells. In one DM1 case (DM1-Patient 1), wherein a comparison with spinal motor neurons was possible, CpG methylation status in spinal motor neurons was relatively low compared with those in cortical neurons and white matter glial cells (Fig. 3). Low CpG methylation status was also observed in spinal motor neurons of another DM1 case (DM1-Patient 4), wherein a measurable amount of DNA was obtained for the methylation analysis. These results indicate that the abnormality of CpG methylation around the CTG repeats was cell-specific in DM1 CNS; cortical neurons, having more unstable repeats, showed higher methylation than white matter glial cells, and spinal motor neurons, having more stable repeats, presented lower methylation status.

### Cell type-specific gene expression profiles in CNS of patients with myotonic dystrophy type 1

**Figure 4:**
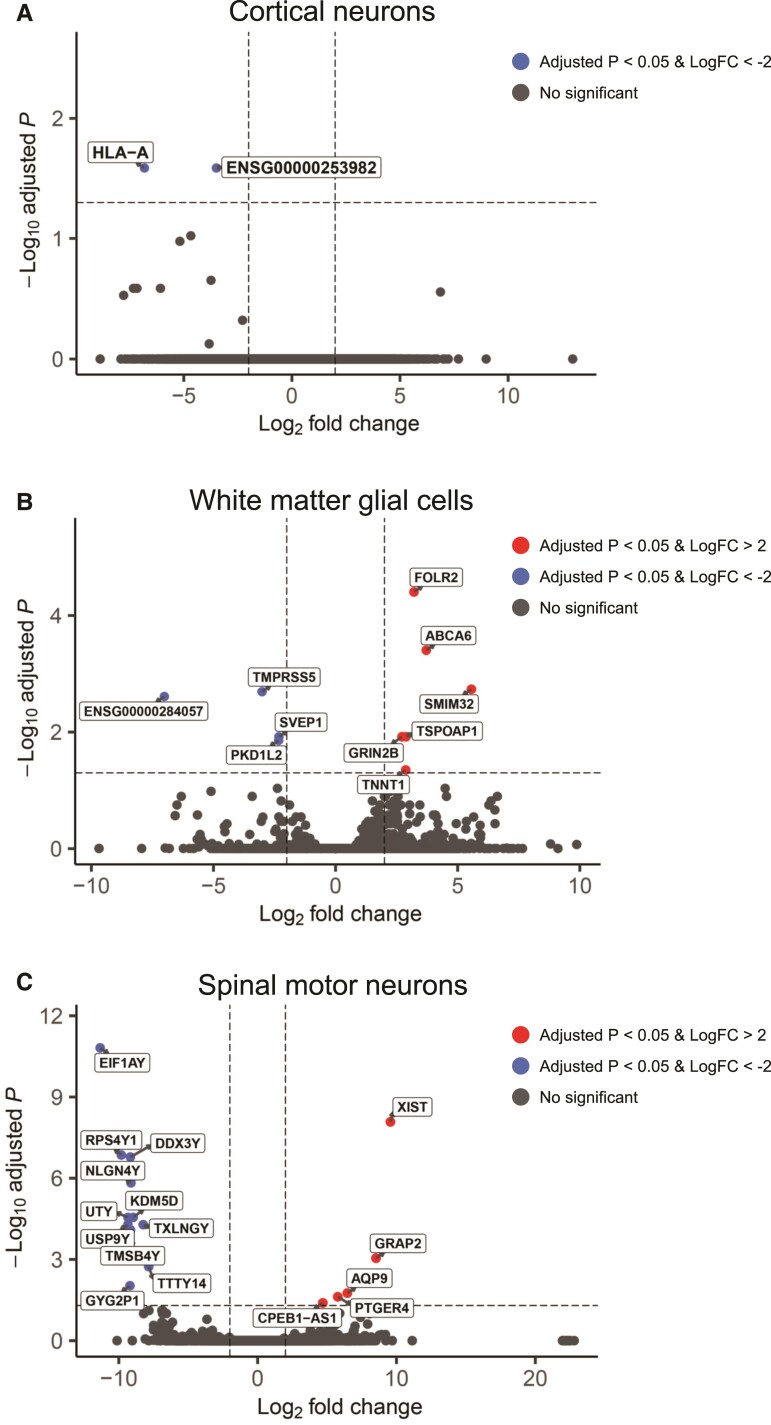
**RNA-seq analysis of gene expression changes in the CNS cell lineages of patients with DM1**. (**A–C**) Volcano plots showing differentially expressed genes in cortical neurons (**A**), white matter glial cells (**B**) and spinal motor neurons (**C**) from patients with DM1 and disease controls (*n* = 3 in each group). Genes with significant enrichment (adjusted *P* < 0.05) are shown as red or blue (log_2_FC >2 or <−2, respectively) across the CNS samples.

To study cell type-specific gene expression profiles in CNS of patients with DM1, we performed RNA-seq analysis using LCM samples obtained from cortical neurons, white matter glial cells and spinal motor neurons from three patients with DM1 and three disease controls. All samples were sequenced to a depth of at least 28 million reads to provide sufficient coverage for the following analyses. The RNA-seq data was mapped (STAR) and quantified (RSEM), and the differential gene expression analysis was performed using DESeq2 (see Materials and methods). Although there was an age and gender bias in the control group, no intra-group bias was found in the transcriptome analysis for either the control or DM1 groups, including the effect of age (Supplementary Fig. 5). The gene expression analysis revealed that only two genes were downregulated and no gene was upregulated in the cortical neurons of patients with DM1 (Fig. 4A, Supplementary Table 3). Similar to the expression profiles of cortical neurons, we found only a few genes with significant changes in expression in the white matter glial cells, such as *GRIN2B*, and spinal motor neurons of patients with DM1 (Fig. 4B and C and Supplementary Table 3). These findings exemplify the difficulty in detecting changes in gene expression using small sample size, characterized by considerable intrinsic CNS cell lineage variability. We also performed GO enrichment analysis with less stringent criteria (*P* < 0.01, |log_2_-fold change| > 2) to clarify the relevant biological pathways (Supplementary Fig. 6). The GO analysis indicated the upregulated and downregulated genes in DM1 cortical neurons were enriched in ‘inflammatory response’ and ‘regulation of angiogenesis,’ respectively (Supplementary Fig. 6A and B). The upregulated genes in DM1 white matter glial cells were enriched in ‘chemical synaptic transmission’ (Supplementary Fig. 6D). To determine whether haploinsufficiency of *DMPK* occurs in DM1 CNS cells, we evaluated the gene expression in each cell lineage. However, the expression level of *DMPK* was not different between DM1 and control groups in each CNS cell lineage (Supplementary Fig. 7A). Previous studies have demonstrated the involvement of DNA repair proteins in repeat instability in repeat expansion disorders, such as DM1.^52^ To examine whether the expression of these genes involved in DNA repair relates to the differences in repeat instability in each CNS cell lineage of DM1, we analyzed gene expression of mismatch repair genes (*MSH2*, *MSH3* and *MSH6*) and *FAN1*. There was no difference between the DM1 and control groups in each CNS cell lineage, but there was a trend toward a lower expression of these genes in the spinal cord than in the cortex and white matter (Supplementary Fig. 7B).

### Cell type-dependent splicing alterations in CNS of patients with myotonic dystrophy type 1

**Figure 5:**
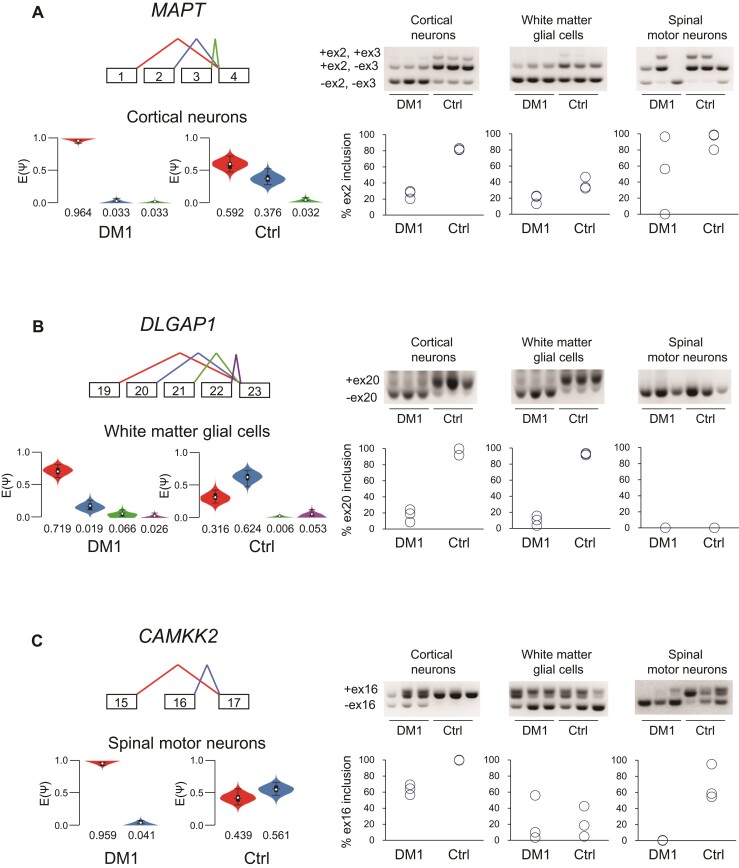
**Cell type-specific splicing alterations in the CNS of patients with DM1**. (**A**) Left: Violin plots depicting the delta PSI of LSVs in *MAPT* gene in cortical neurons. Right: Representative gel images of RT-PCR products for each CNS cell lineage (neuronal cells, glial cell and motor neurons) from patients with DM1 and disease controls (top). The percentage of *MAPT* exon 2 inclusion in each sample (bottom). (**B**) Left: Violin plots depicting the delta PSI of LSVs in *DLGAP1* gene in white matter glial cells. Right: Representative gel images of RT-PCR products (top). The percentage of *DLGAP1* exon 20 inclusion in each sample (bottom). (**C**) Left: Violin plots depicting the delta PSI of LSVs in *CAMKK2* gene in spinal motor neurons. Right: Representative gel images of RT-PCR products (top). The percentage of *CAMKK2* exon 16 inclusion in each sample (bottom). Uncropped gels are shown in Supplementary Fig. 10.

Next, we analyzed the RNA-seq data set using MAJIQ and Voila software^41^ to determine cell type-dependent splicing alterations in the CNS of patients with DM1. We quantified LSVs and measured the relative LSV abundance [change in percentage selected index (dPSI)] between patients with DM1 and disease controls. This analysis identified 176 LSVs that were differentially spliced in cortical neurons from patients with DM1 and disease controls at an estimated dPSI [E(dPSI)] threshold of 0.1 and a confidence threshold of 0.95 (Supplementary Table 4). The LSVs with high E(dPSI) value (>0.3) consisted of several misspliced exons that were previously identified in the DM1 brain (*MAPT* exon 2, *MBNL2* exon 8 and *CLASP2* exon 23),^16,19,53^ indicating the validity of our analysis (Table 2, Fig. 5A and Supplementary Fig. 8A). Alternative splicing events with high E(PSI) included the *ADD3* exon 16, missplicing of which was reported in DM1 heart,^7^ in addition to newly identified missplicing events, such as *LMO7* exon 39 and *RABGAP1* exon 19 (Table 2 and Supplementary Fig. 8B).

**Table 2:** Mean dPSI values for selected genes with significant splicing differences between patients with DM1 and disease controls in each CNS cell lineage determined by MAJIQ

Cortical neurons, E(dPSI) >0.3		White matter glial cells, E(dPSI) >0.3		Spinal motor neurons, E(dPSI) >0.3	
Gene	Mean dPSI	Gene	Mean dPSI	Gene	Mean dPSI
***CLASP2***	0.600	***AC093330.1***	0.971	***TVP23C-CDRT4***	0.859
***ZFP82***	0.605	***GUSBP1***	0.936	***ABHD12***	0.762
***AL390719.1***	0.590	***GUSBP1***	0.789	***DPY19L1P1***	0.739
***LMO7***	0.548	***SNHG17***	0.669	***TVP23C***	0.705
***MAPT***	0.541	***ITGB2***	0.570	***PPP6R2***	0.612
***RABGAP1***	0.526	***LACC1***	0.435	***AC008105.1***	0.591
***L3HYPDH***	0.525	***DLGAP1***	0.426	***TOX3***	0.588
***AP001347.1***	0.514	***AC018521.1***	0.415	***TREX1***	0.578
***SH3KBP1***	0.503	***AVPI1***	0.418	***RNF216P1***	0.575
***ADAL***	0.500	***MAGI2***	0.410	***WNK1***	0.570
***RNPS1***	0.481	***NTM***	0.409	***TNS1***	0.563
***MEG3***	0.475			***ZCWPW1***	0.545
***ADD3***	0.423			***OSER1-DT***	0.541
				***GIT2***	0.538
				***CNTNAP3B***	0.537
				***CAMKK2***	0.528
				***MPDZ***	0.526
				***ITGB2***	0.506
				***FRS2***	0.503
				***CENPS-CORT***	0.503
				***SERPINA1***	0.500
				***MINK1***	0.498
				***FAM228B***	0.495
				***PEAK1***	0.495
				***ZFAND6***	0.479
				***EPB41L2***	0.478
				***TK1***	0.468
				***RIPK2***	0.465
				***LINC00323***	0.462
				***PARP2***	0.461
				***EPB41L2***	0.458
				***FRYL***	0.446

DM1, myotonic dystrophy type 1; dPSI, change in percentage selected index.

Most of the previous splicing studies in DM1 brain were conducted using the cortex tissues. Because the characteristic neuroimaging and histological features of DM1 are also observed in the white matter,^21,54^ we investigated splicing misregulation in glial cells, which are the major cellular constituent of white matter. We identified 80 LSVs differentially regulated at an E(dPSI) threshold of 0.1 and a confidence threshold of 0.95 (Supplementary Table 4). The LSVs with high E(dPSI) value (>0.3) included *DLGAP1* exon 20, *NTM* exon 21 and *ITGB2* exon 11 (Table 2, Fig. 5B and Supplementary Fig. 8C). Then, we validated the aberrant splicing events in each sample via RT-PCR. The splicing abnormalities detected in the white matter tended to be larger in the white matter glial cells than in the cortical neurons (Fig. 5B and Supplementary Fig. 8C).

Furthermore, we studied splicing misregulation in spinal motor neurons of patients with DM1, which had never been investigated before. We found 375 LSVs differentially regulated at an E(dPSI) threshold of 0.1 and a confidence threshold of 0.95 (Supplementary Table 4). The LSVs with high E(dPSI) value (>0.3) included *CAMKK2* exon 16 and *WNK1* exon 12 (Table 2, Fig. 5C and Supplementary Fig. 8D). The splicing abnormalities identified in the spinal motor neurons tended to be more prominent in the spinal motor neurons than in the other CNS cells (Fig. 5C and Supplementary Fig. 8D), suggesting the degree of splicing abnormalities in DM1 varies among the CNS cell lineages.

### Aberrant *CAMKK2* splicing and neurite morphology in myotonic dystrophy type 1 motor neurons

**Figure 6:**
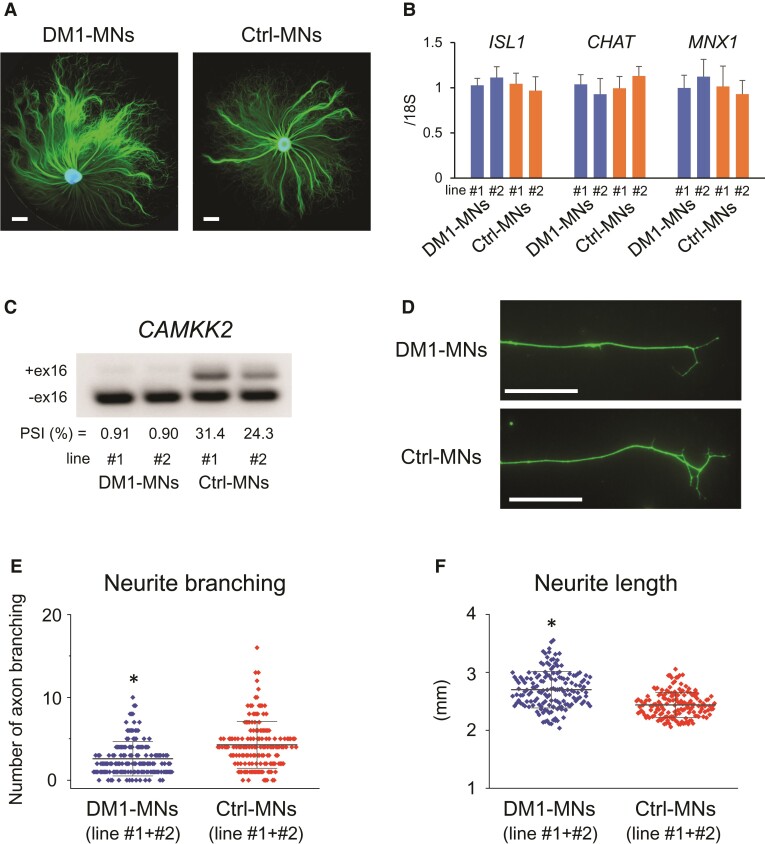
**Missplicing of *CAMKK2* in DM1 motor neurons associated with changes in neurite elongation/branching**. (**A**) Immunostaining for the neuronal marker TUJ1 (green) at 8 days post-plating (DPP) in motor neurons differentiated from iPSCs derived from a patient with DM1 (DM1-MNs line #1) and a healthy individual (Ctrl-MNs line #1). Nuclei are stained with Hoechst 33342. Scale bars represent 1 mm. (**B**) The expression of motor neuron markers was not different between DM1-MNs (lines #1 and #2) and Ctrl-MNs (lines #1 and #2). (**C**) Representative gel images of RT-PCR products of *CAMKK2* in DM1-MNs (lines #1 and #2) and Ctrl-MNs (lines #1 and #2). Uncropped gels are shown in Supplementary Fig. 10. (**D**) Representative images of the axon end with branching in DM1-MNs and Ctrl-MNs. Scale bars represent 100 μm. (**E**) The axon branching was decreased in DM1-MNs (lines #1 and #2) compared with Ctrl-MNs (lines #1 and #2). The largest 10 axons were analyzed in each of eight biological replicates (160 axons in each group). Data are shown as means ± standard deviation. * *P* < 0.0001, two-tailed Student’s *t*-test. The comparisons of the average number of axon branching in each biological replicate are shown in Supplementary Fig. 9. (**F**) The axon length was increased in DM1-MNs (lines #1 and #2) compared with Ctrl-MNs (lines #1 and #2). The largest 10 axon lengths were analyzed in each of eight biological replicates (160 axons in each group). Data are shown as means ± standard deviation. * *P* < 0.0001, two-tailed Student’s *t*-test. The comparisons of the average axon length in each biological replicate are shown in Supplementary Fig. 9.

Then, we investigated the association between aberrant splicing and phenotypic changes in motor neurons of patients with DM1. We focused on exon 16 of *calcium/calmodulin dependent protein kinase kinase 2 (CAMKK2)*, a gene that plays an essential role in neuronal differentiation.^55^ The differential splicing of exon 16 affects the ability of CAMKK2 to control axon formation: a splicing variant lacking exon 16 promotes neurite elongation, whereas another variant containing exon 16 promotes neurite branching.^56^ To evaluate the phenotype of neurite formation, we differentiated hiPSCs derived from two patients with DM1 and two healthy individuals into motor neurons (DM1-MNs lines #1 and #2 and Ctrl-MNs lines #1 and #2, respectively, Fig. 6A). No change in the expression of motor neuron markers (*ISL1*, *CHAT* and *MNX1*) was observed in DM1 motor neurons (DM1-MNs lines #1 and #2) compared with control motor neurons (Ctrl-MNs lines #1 and #2) (Fig. 6B). The expression of *MBNL1* and *MBNL2* was not different between DM1-MNs and Ctrl-MNs (Supplementary Fig. 7C). The *CAMKK2* variant containing exon 16, which promotes neurite branching and suppresses neurite elongation,^56^ was decreased in DM1-MNs lines #1 and #2, similar in spinal motor neurons of patients with DM1 (Fig. 6C). In accordance with the splicing shift towards the *CAMKK2* variant lacking exon 16, we observed a significantly lower degree of axon branching in DM1-MNs lines #1 and #2 than in Ctrl-MNs lines #1 and #2 (*P* = 4.92 × 10^−9^; Fig. 6D and E and Supplementary Fig. 9). We also found enhanced neurite outgrowth in DM1-MNs lines #1 and #2 (*P* = 3.08 × 10^−16^; Fig. 6F and Supplementary Fig. 9). These results suggest the possibility that aberrant splicing of *CAMKK2* exon 16 in DM1 affects axon morphology by blocking branching formation.

## Discussion

Despite the name ‘myotonic dystrophy,’ non-muscular symptoms of DM1 have as much impact on patients as skeletal muscle and heart symptoms.^3,4^ Patients with DM1 present with various CNS symptoms, such as cognitive and concentration disturbances, reduced attention and flexibility of thinking and excessive daytime sleepiness. However, the pathogenesis of these CNS symptoms is largely unknown. Previous studies investigated the pathomechanism of CNS symptoms and provided clues—neuropathological studies showed neuronal loss in the cortex and myelin loss in the white matter of patients with DM1.^21^ Neuroimaging studies demonstrated white matter hyperintensity in the frontal and temporal lobes, especially in the anterior temporal pole,^22^ and neurophysiological studies revealed the presence of axonal neuropathy in patients with DM1.^48–50^ Based on these previous findings, we targeted individual CNS cells in specific regions (anterior temporal pole and spinal anterior horn) and conducted molecular analysis on cortical neurons, white matter glial cells and spinal motor neurons using an LCM-based approach.

First, our findings revealed differences in somatic repeat instability among the different CNS cell lineages of patients with DM1. CTG repeat length was more unstable and longer in cortical neurons than in white matter glial cells in the DM1 brain. This observation is similar to the findings of a previous study that reported longer CTG repeat length in cerebral grey matter than in the white matter of patients with DM1, although cell types were not discriminated in the study.^57^ Neurons tend to have more unstable and longer CAG repeat length than glial cells in patients with Huntington’s disease.^58^ In contrast, another study reported that expanded CAG repeat length in cerebral neuronal cells is shorter than in cerebral glial cells of patients with dentatorubral-pallidoluysian atrophy.^59^ Therefore, somatic repeat instability varies in neuronal and glial cells in patients with DM1 and other repeat expansion disorders, and the degree of repeat instability among these CNS cells depends on the genetic locus of expanded repeats. Moreover, we revealed that CTG repeat length was very stable and short in spinal motor neurons of patients with DM1. In DM1, the repeat length in the cerebellum is also short.^25,31,57^ These findings suggest the involvement of unknown cell type-specific trans-factor in repeat instability in the CNS cell lineages of DM1. Indeed, the expression levels of DNA repair genes involved in repeat instability tended to be lower in the spinal cord than in the cortex and white matter. Interestingly, although the CTG repeats in DM1 spinal motor neurons were stable, the length was considerably longer than the estimated progenitor allele length (Table 1). This discrepancy suggests a saltatory process that creates large expansions in a single step during early development rather than steady accrual of small expansion during age.

Several previous studies reported hypermethylation of CpG sites upstream of expanded CTG repeats in DM1 tissues.^25,33^ Moreover, CpG methylation status showed tissue-specific differences in DM1 and higher methylation levels in the cerebral cortex.^25^ We observed variation in CpG methylation from each CNS cell lineage even in the same patient; cortical neurons show higher methylation, and spinal motor neurons present less methylation. CpG methylation around the repeats and binding with the insulator protein CTCF could regulate CTG repeat instability.^25^ In particular, aberrant CpG methylation at CTCF binding site upstream of the repeats is associated with repeat length in CDM muscles.^33^ Our results also indicate a possible correlation between methylation status and repeat instability in CNS cells of DM1 (*i.e*., greater repeat instability in cortical neurons and less instability in spinal neurons). However, the detailed mechanism of how expanded repeats induce CpG methylation around the repeat tract, or vice versa, remains unknown.

We also investigated gene expression profiles in each CNS cell lineage obtained via LCM. Only a few genes were differentially expressed in CNS cells from patients with DM1, possibly due to the small number and gender bias of samples. However, we found significant upregulation of a gene encodes for the NR2B subunit of the N-methyl-D-aspartate (NMDA) receptor—*GRIN2B*— in white matter glial cells of patients with DM1. As a glial expression of the NMDA receptor containing NR2B is induced by anoxia as a part of neuroprotective response,^60^ the upregulation of *GRIN2B* in DM1 glial cells may reflect its neuroprotective effect in the white matter of patients with DM1. Compared with previous transcriptomic studies in DM1, the differentially expressed genes in DM1 CNS cells did not overlap with transcriptional signatures associated with brain volume loss or neuropsychological deficits in DM1.^61^ The differentially expressed genes in DM1 CNS cells did not overlap with those found in RNA-seq analysis for DM1 frontal cortex.^8^ However, GO analysis showed upregulated genes in DM1 cortical neurons were significantly related to ‘inflammatory response,’ which were enriched in upregulated genes in the DM1 frontal cortex in the previous study. Interestingly, significant upregulation of inflammatory response genes was reported in CDM muscles,^33^ indicating the proinflammatory effect caused by toxic RNAs in DM1.

Abnormal regulation of alternative splicing caused by toxic RNAs is a molecular hallmark of DM1.^6^ Splicing defects in various genes have been identified in DM1 muscles, and some are suggested to cause muscular symptoms.^7,9,10,12,13,15^ A handful of missplicing events have also been reported in DM1 brains, mostly in the cortex.^8,16–18^ This study identified splicing misregulation of a series of genes in cortical neurons of patients with DM1, obtained via LCM. Some of the splicing misregulation, such as *MAPT* exon 2 and *CLASP2* exon 23, substantially overlap previously reported missplicing events, consistent with the fact that the previous studies were performed using cortical samples. Aberrant splicing of *MAPT* exon 2 is the first reported splicing abnormality in the DM1 brain and has been suggested to be involved in NFTs formation, which is often observed in the temporal and frontal cortices of patients with DM1.^16,62^ CLASP2 is suggested to play an important role in neuronal differentiation, although the biological significance of abnormal splicing of *CLASP2* exon 23 is unknown.^63^ The aberrant splicing in DM1 cortical neurons detected in our study may be involved in the neuronal loss in the cerebral cortex reported in previous neuropathological studies. Our analysis also focused on splicing abnormalities in the white matter, wherein the characteristic neuropathological and neuroradiological abnormalities are present in patients with DM1.^21,22^ We found many misregulated splicing events in the white matter glial cells of patients with DM1. Among these events, missplicing of *DLGAP1* exon 20 has been reported in the frontal cortex of patients with DM1.^8^ However, in our study, the splicing abnormality in *DLGAP1* exon 20 was more prominent in glial cells than in the cortical neurons of patients with DM1, similar to other missplicing events identified in DM1 glial cells, such as *NTM* exon 21. These results indicate that splicing abnormality in DM1 brains is not always more extensive in the cortical neurons compared with the glial cells. Although misregulated splicing of myelin-related genes was not identified in our analysis, splicing abnormality specific to white matter glial cells may be involved in the myelin loss and WMHI demonstrated in previous studies on DM1.

Thus far, abnormal splicing in the CNS of DM1 has been examined only in the brain. However, many neurophysiological and histological studies pointed to the involvement of spinal motor neurons in DM1 pathogenesis.^46–50^ Clinically, peripheral neuropathy in phrenic motor neurons affects the respiratory function of patients with DM1.^64^ Abnormal morphological motor nerve terminals were also reported in patients with DM1 and DM1 model mice.^65,66^ In this study, we investigated the pathomechanism of the defects in motor neurons and found a number of missplicing events in the spinal motor neurons of patients with DM1. As is the case with *DLGAP1* exon 20 in the white matter glial cells, the splicing abnormality in these events, such as *CAMKK2* exon 16, was more apparent in the DM1 spinal motor neurons compared with the other CNS cells or not apparent in the other CNS cells, which also indicates the cell type-dependent variation of splicing misregulation in DM1. CAMKK2 is one of the most versatile calmodulin kinases and regulates neuronal development.^55^ The alternative splicing of *CAMKK2* exon 16 results in a functional difference in the protein between axonal elongation and branching.^56^ In our study, the *CAMKK2* variant containing exon 16, which leads to axon branching, was decreased in DM1 spinal motor neurons. Motor neurons differentiated from both lines of DM1 iPSCs showed similar splicing misregulation in *CAMKK2* and relevant phenotypic changes—decreased axonal branching and enhanced neurite elongation. A previous study using motor neurons differentiated from DM1 embryonic stem cells also demonstrated abundant neuritogenesis associated with a marked impairment in the formation of neuromuscular connections.^67^ Because reduced axon branching is associated with abnormal neuromuscular junction formation,^68^ our results suggest the possibility that the aberrant splicing of *CAMKK2* in DM1 affects the morphology of motor neurons by altering the balance between neurite branching and elongation, although how the decreased axon branching induces DM1 pathology remains unclear. It is also unclear whether the missplicing of *CAMKK2* and morphological abnormalities found in DM1 iPSC-derived motor neurons model impaired neuritogenesis during development or axonal maintenance in adulthood. Furthermore, to prove a direct causative link between missplicing of *CAMKK2* and abnormal motor neuron morphology, future studies using inducible CRISPR-Cas system or antisense oligonucleotides-based exon skipping to restore the normal splicing of *CAMKK2* will be needed, although both approaches are currently difficult for highly differentiated iPSC-derived motor neurons.

In our study, to elucidate the CNS pathogenesis in patients with DM1, we analyzed each individual CNS cell lineage using an LCM-based approach. A major limitation of this study was the sample size available for analyses. We restricted the study population to patients with DM1 whose pathological specimens of cortex and white matter in the anterior temporal pole were both available and brain MRI showed the WMHI; therefore, we could only study three cases with DM1 in each CNS lineage with considerable intrinsic variability. Furthermore, due to the difficulty in obtaining spinal cord samples, comparisons with cortical neurons and white matter glial cells in the same patient with DM1 could not be made in all cases. Indeed, because of the paucity of the spinal cord samples, there have been very few studies on the pathogenesis in the spinal cord of patients with DM1 until now, with only one paper reporting ribonuclear foci in spinal motor neurons.^46^ Nonetheless, we found that CTG repeat length and instability, abnormal CpG methylation and splicing abnormalities differ among the cortical neurons, white matter glial cells and spinal motor neurons of patients with DM1. We also revealed the splicing abnormalities in the white matter and spinal motor neurons of patients with DM1, which have not received much attention but might have significant impact on characteristic neuropathological, neuroimaging and neurophysiological CNS features in DM1. Further elucidation of the CNS pathogenesis in each cell lineage, especially detailed studies using single-cell analysis with larger sample sizes, will reveal the causes of CNS symptoms in patients with DM1 and support the development of therapies for CNS manifestation.

## Supplementary Material

fcac154_Supplementary_Data

## Data Availability

Sequencing data have been deposited in Gene Expression Omnibus under accession number GSE 198321.

## Abbreviations

CDM =: congenital DM1
CNS =: central nervous system
DM1 =: myotonic dystrophy type 1
hiPSCs =: human-induced pluripotent stem cells
LCM =: laser-capture microdissection
LSV =: local splice variation
MN =: motor neuron
UTR =: untranslated region
WMHI =: white matter hyperintense lesions
