# Supplementary material for: Cell type-specific abnormalities of central nervous system in myotonic dystrophy type 1
Source: Brain Commun. 2022 Jun 10;4(3):fcac154. doi: 10.1093/braincomms/fcac154 (PMC9218787; doi:10.1093/braincomms/fcac154)
Supplement: fcac154_Supplementary_Data [file fcac154_supplementary_data.zip › Supplementary Figures_final.pdf]

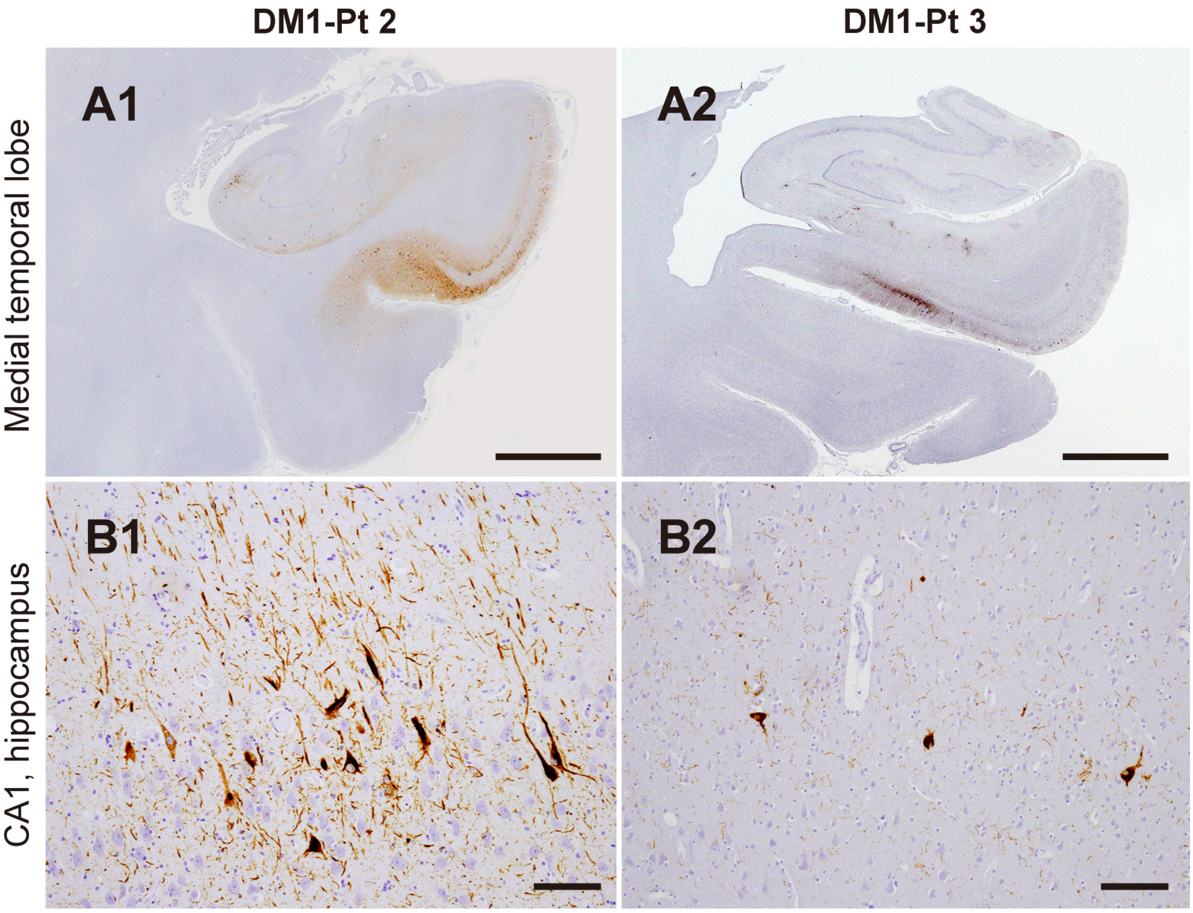

Supplementary Figure 1. Immunohistochemical examination of phosphorylated tau in patients with DM1.

(A1, B1) DM1-Patient 2. Phosphorylated tau (AT8)-positive neurofibrillary tangles (NFTs) and neurites are abundant in the parahippocampal cortex **(A1)** and hippocampus **(B1)**, with a distribution pattern corresponding to Braak stage II. **(A2, B2)** DM1-Patient 3. AT8-positive NFTs and neurites were virtually confined to the entorhinal cortex **(A2)** and sparse in the CA1 subfield of the hippocampus **(B2)**, with a distribution pattern corresponding to Braak stage I. Bars indicate 5 mm for **A1** & **A2** and 100 μm for **B1** & **B2**.

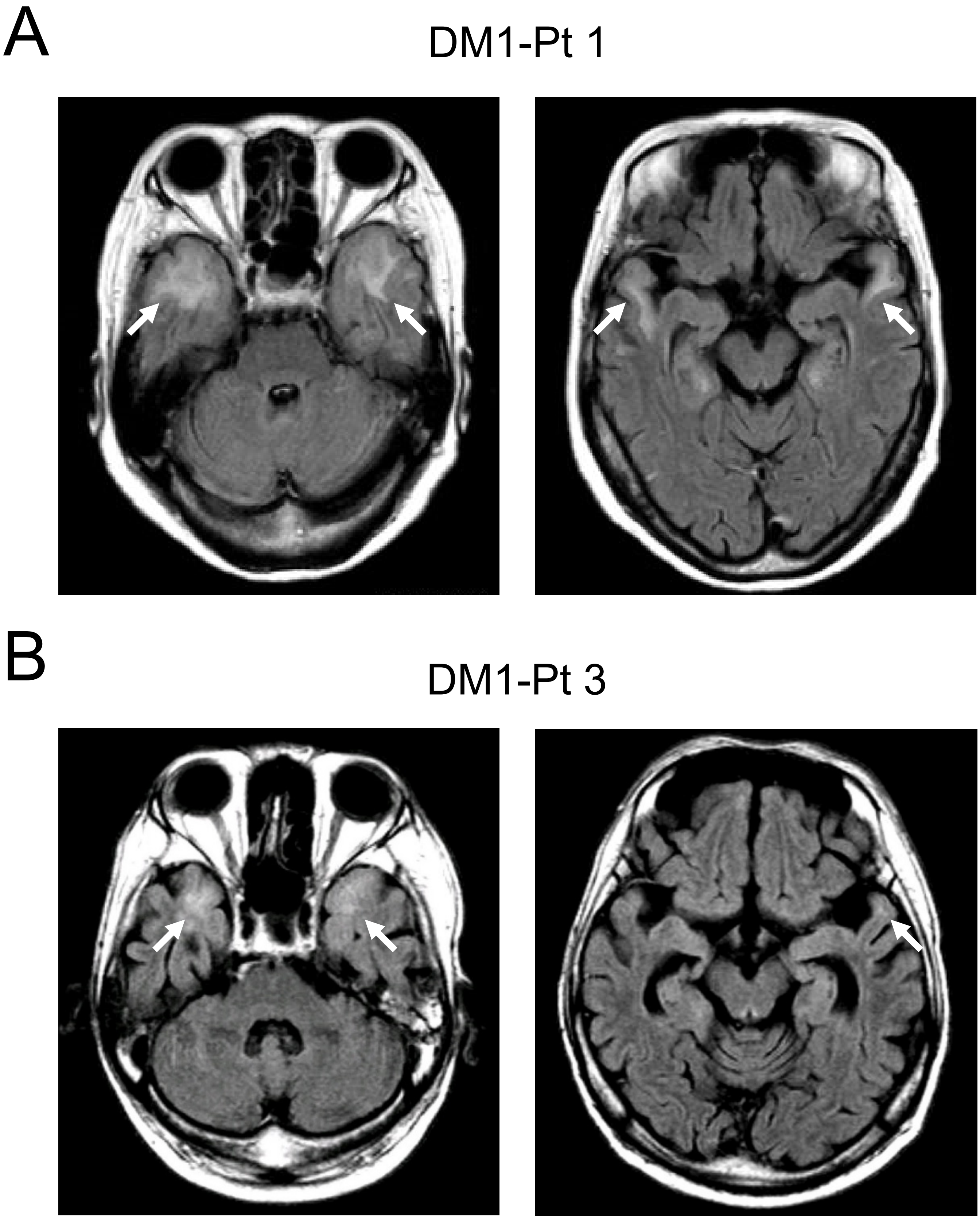

Supplementary Figure 2. Brain MRI images of patients with DM1.
Brain MRI fluid-attenuated inversion recovery (FLAIR) axial images showing bilateral white matter hyperintense lesions (white arrows) in the anterior temporal pole of DM1-patients 1 (DM1-Pt 1) **(A)** and 3 (DM1-Pt 3) **(B)**.

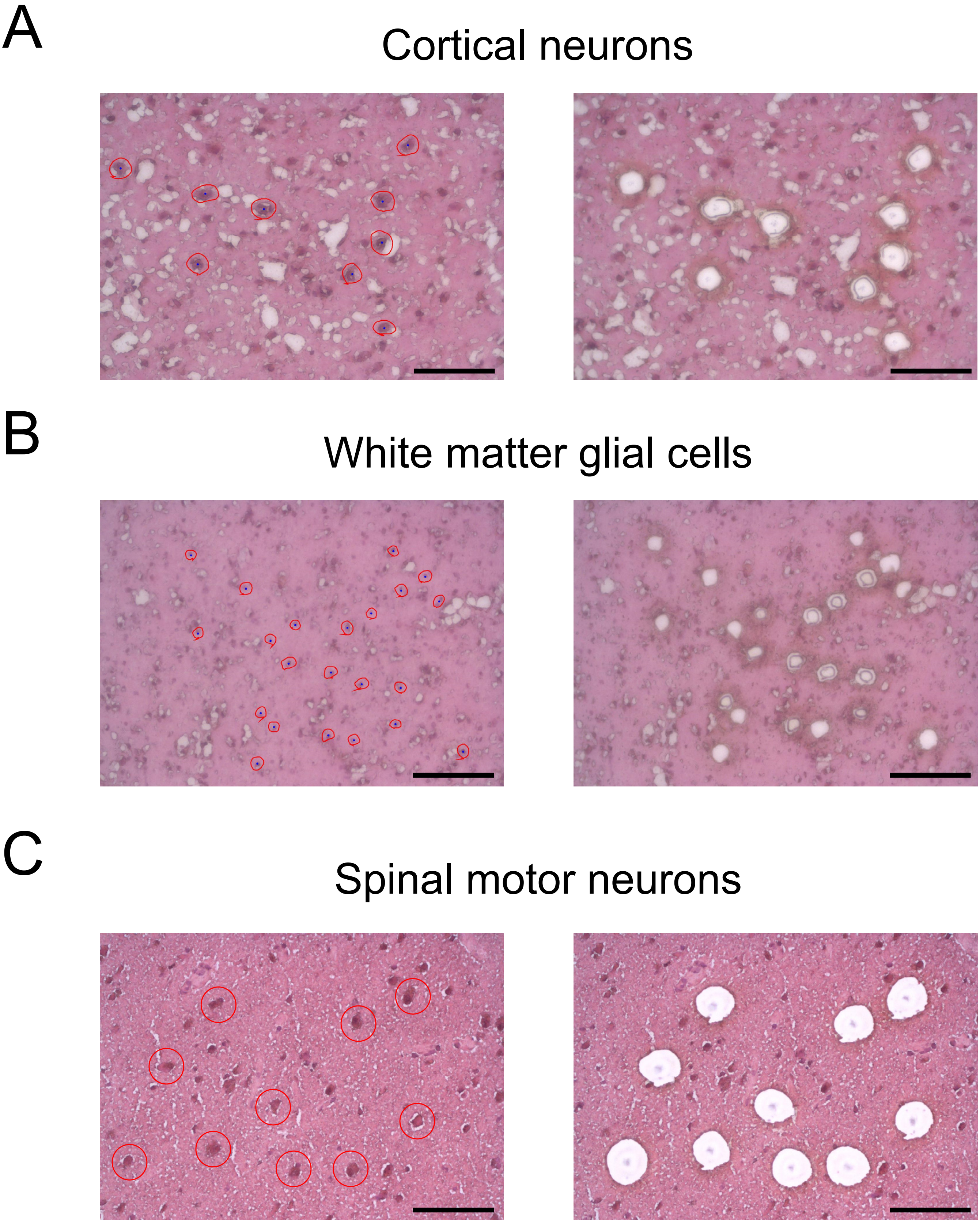

Supplementary Figure 3. Representative images of LCM of cortical neuronal cells **(A)**, white matter glial cells **(B)**, and spinal motor neurons **(C)**. Areas circled in red were dissected via LCM. Bars: 100 µm.

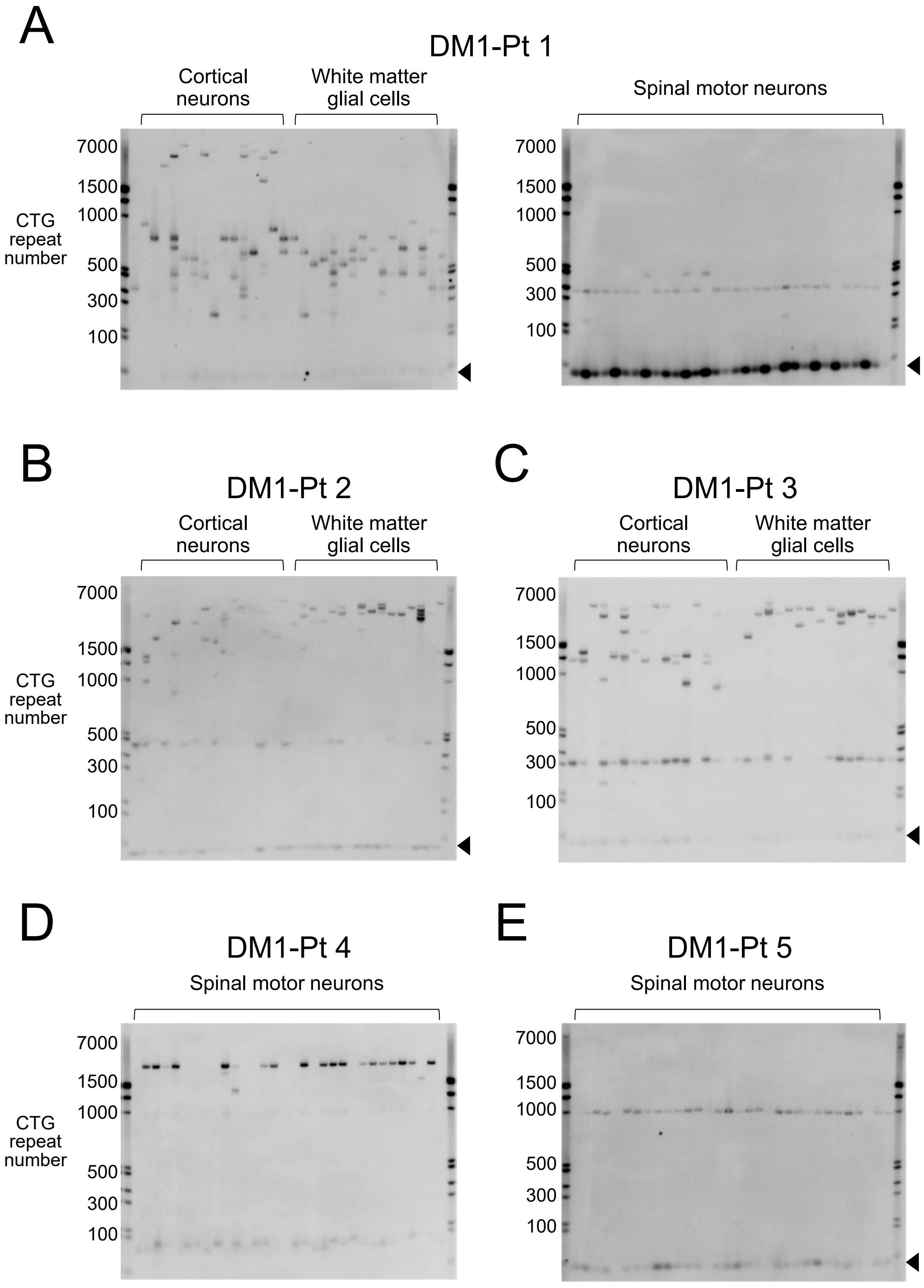

Supplementary Figure 4. Small-pool PCR analysis of repeat length in the CNS cells of patients with DM1. Representative lanes of small-pool PCR are shown. Arrow heads indicate normal *DMPK* alleles. **(A)** Repeat instability in cortical neurons, white matter glial cells, and spinal motor neurons from DM1-patient 1 (DM1-Pt 1). **(B)** Repeat instability in cortical neurons and white matter glial cells from DM1-patient 2 (DM1-Pt 2). **(C)** Repeat instability in cortical neurons and white matter glial cells from DM1-patient 3 (DM1-Pt 3). **(D)** Repeat instability in spinal motor neurons from DM1-patient 4 (DM1-Pt 4). **(E)** Repeat instability in spinal motor neurons from DM1-patient 5 (DM1-Pt 5).

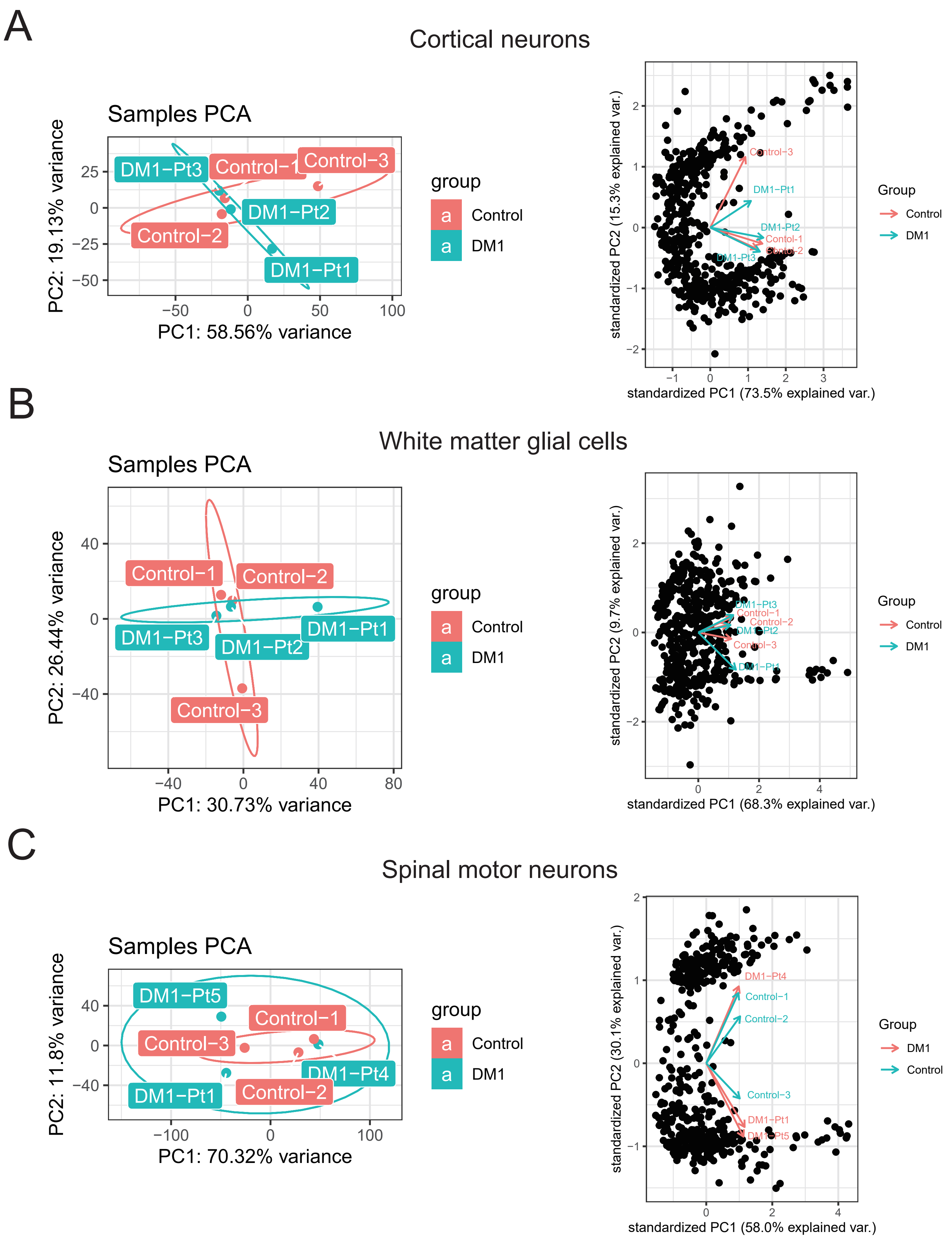

Supplementary Figure 5 . Principal components analysis (PCA) on the top 500 most variable genes identified by transcript profiling.

(A) A sample PCA plot (left) and a biplot display (right) on a data set of cortical neurons from DM1 and control groups. **(B)** A sample PCA plot (left) and a biplot display (right) on a data set of white matter glial cells from DM1 and control groups. **(C)** A sample PCA plot (left) and a biplot display (right) on a data set of spinal motor neurons from DM1 and control groups.

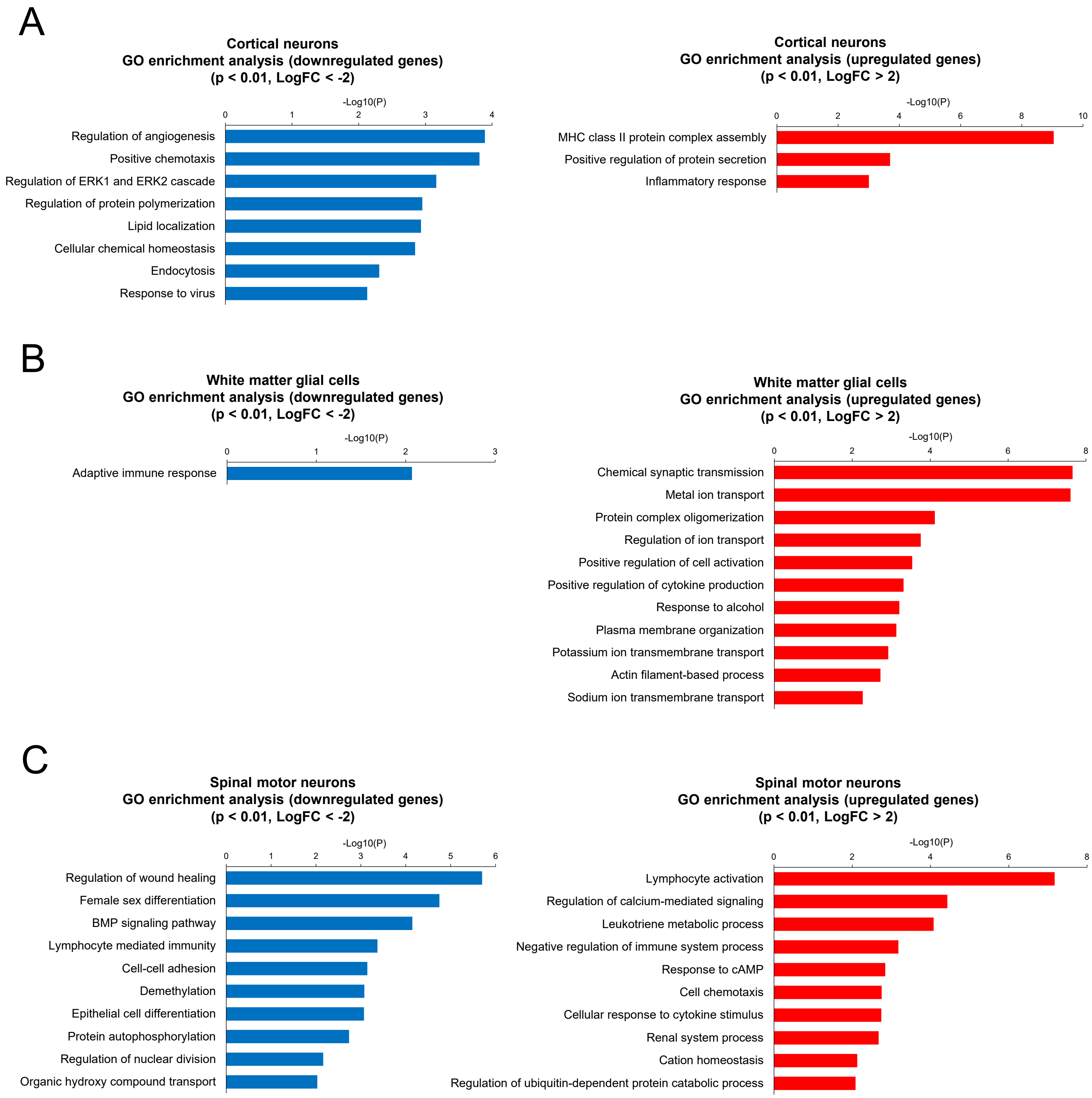

Supplementary Figure 6. RNA-seq analysis in the CNS cells of patients with DM1.
(A) Bar graphs displaying the gene ontology (GO) categories showing significant enrichment for downregulated (left, blue) and upregulated (right, red) genes in DM1 cortical neurons. **(B)** Bar graphs displaying the GO categories showing significant enrichment for downregulated (left, blue) and upregulated (right, red) genes in DM1 white matter glial cells. **(C)** Bar graphs displaying the GO categories showing significant enrichment for downregulated (left, blue) and upregulated (right, red) genes in DM1 spinal motor neurons.

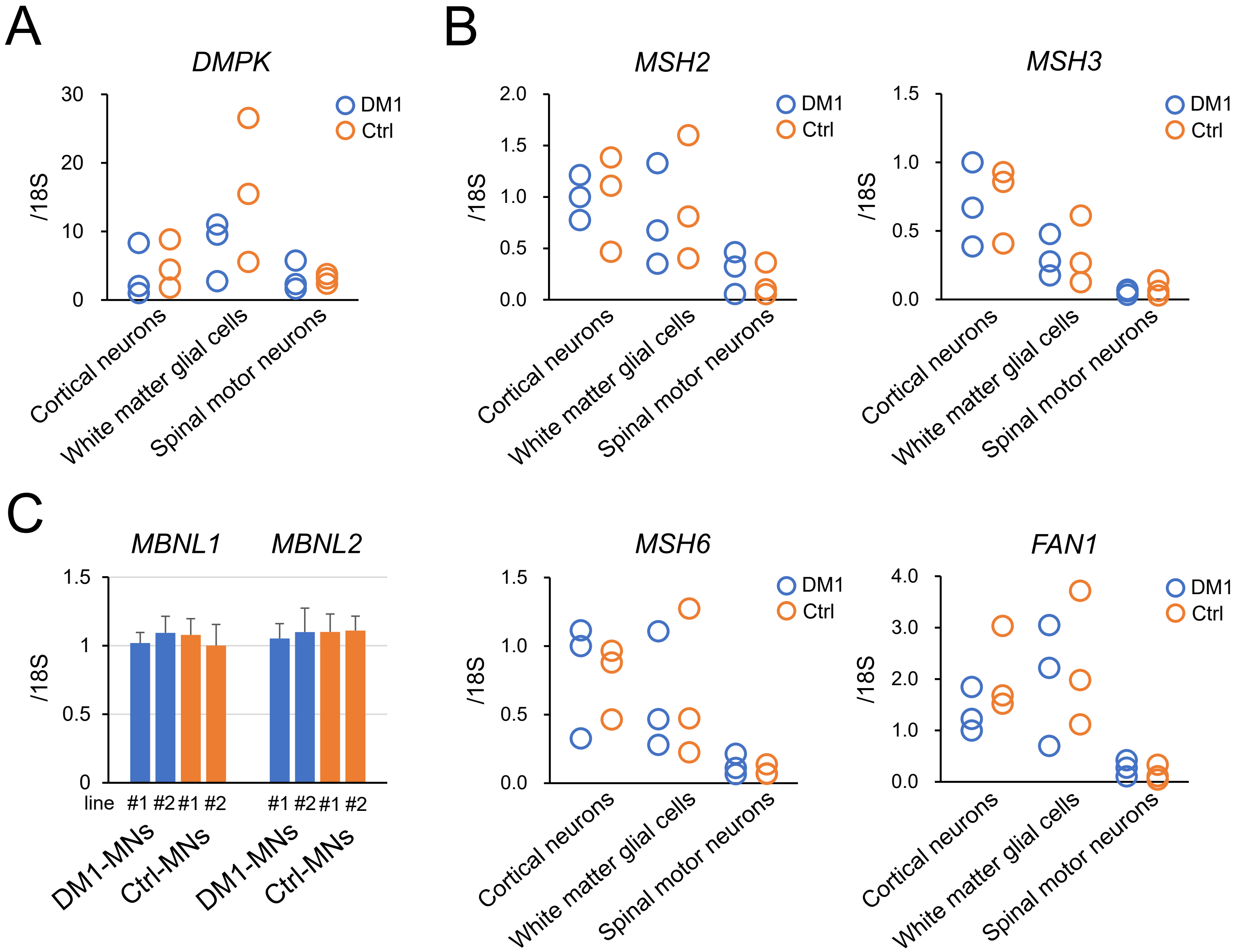

Supplemental Figure 7. (**A**) Expression levels of *DMPK* genes in CNS cells in DM1 as determined by *18S* rRNA-normalized quantitative reverse transcription PCR. **(B)** Expression levels of mismatch repair genes (*MSH2*, *MSH3*, and *MSH6*) and *FAN1* in CNS cells in DM1. **(C)** The expression of *MBNL1* and *MBNL2* was not different between DM1-MNs (lines #1 and #2) and Ctrl-MNs (lines #1 and #2).

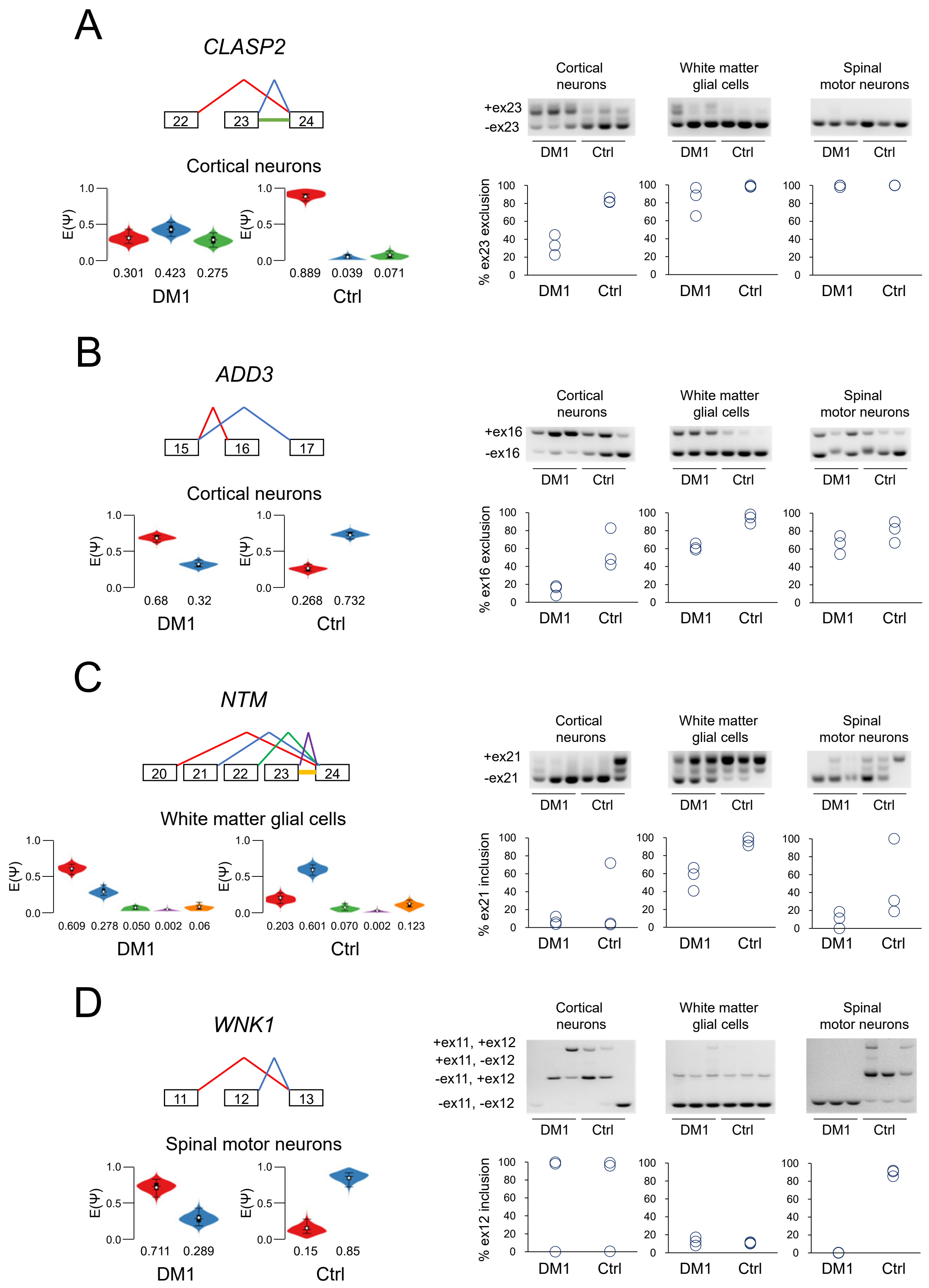

Supplementary Figure 8. Cell type-specific splicing alterations in the CNS of patients with DM1.
(A) Left: Violin plots depicting the delta PSI of LSVs in *CLASP2* gene in cortical neuronal cells. Right: Representative gel images of reverse transcriptase PCR (RT-PCR) products are shown for each of the CNS cell lineage (neuronal cells, glial cell, and motor neurons) from patients with DM1 and disease controls (top). The percentage of *CLASP2* exon 23 exclusion in each sample (bottom). **(B)** Left: Violin plots depicting the delta PSI of LSVs in *ADD3* gene in cortical neurons. Right: Representative gel images of RT-PCR products (top). The percentage of *ADD3* exon 16 exclusion in each sample (bottom). **(C)** Left: Violin plots depicting the delta PSI of LSVs in *NTM* gene in the white matter glial cells. Right: Representative gel images of RT-PCR products (top). The percentage of *NTM* exon 21 inclusion in each sample (bottom). **(C)** Left: Violin plots depicting the delta PSI of LSVs in *WNK1* gene in spinal motor neurons. Right: Representative gel images of RT-PCR products (top). The percentage of *WNK1* exon 12 inclusion in each sample (bottom). Uncropped gels are shown in Supplementary Figure 10.

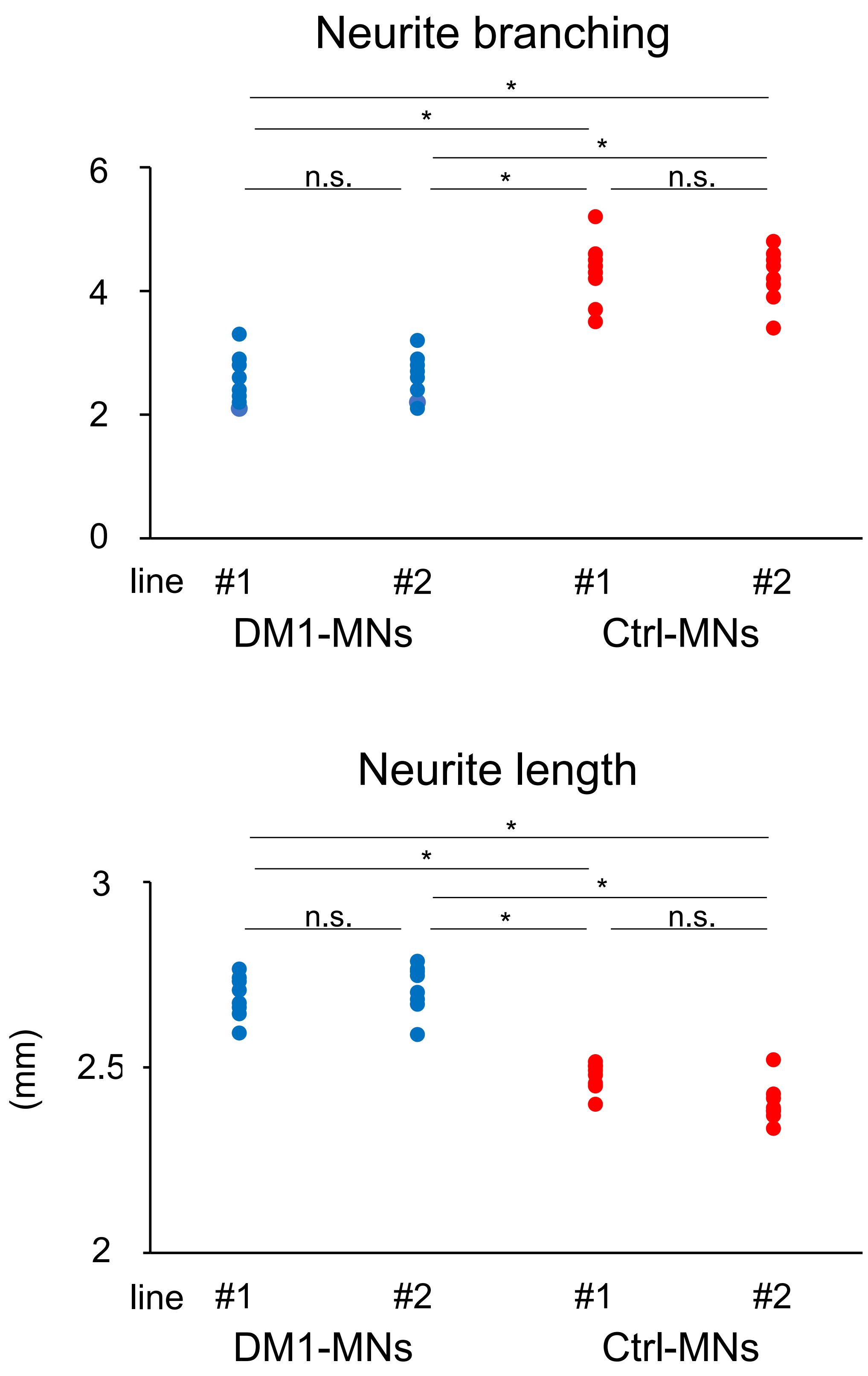

Supplementary Figure 9. Neurite elongation/branching in motor neurons derived from each line of iPSCs.

Top: The average number of axon branching in each biological replicate of motor neurons derived from iPSCs (DM1-MNs lines #1 and #2, and Ctrl-MNs lines #1 and #2). Bottom: The average axon length in each biological replicate of motor neurons derived from iPSCs (DM1-MNs lines #1 and #2, and Ctrl-MNs lines #1 and #2). Two-way ANOVA with post hoc Tukey HSD tests for among each cell line. n.s. = not significant, *$p < 0.0001$.

Figure 5A

Cortical neurons

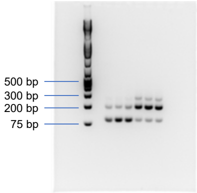

White matter glial cells

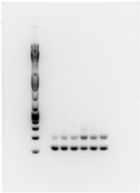

Spinal motor neurons

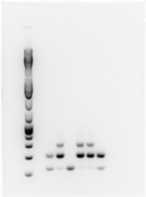

Figure 5B

Cortical neurons

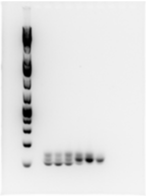

White matter glial cells

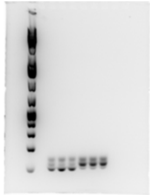

Spinal motor neurons

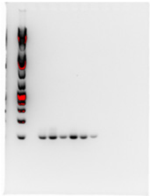

Figure 5C

Cortical neurons

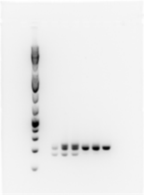

White matter glial cells

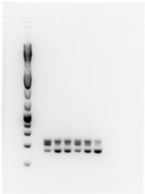

Spinal motor neurons

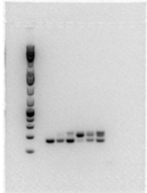

Figure 6C

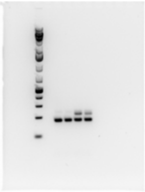

Supplementary Figure 8A

Cortical neurons

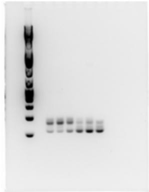

White matter glial cells

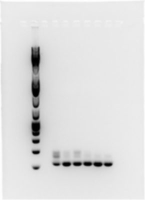

Spinal motor neurons

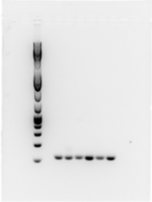

Supplementary Figure 8B

Cortical neurons

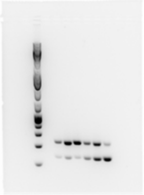

White matter glial cells

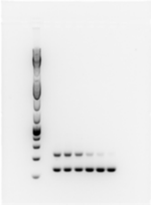

Spinal motor neurons

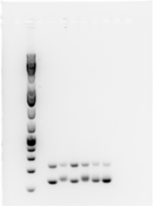

Supplementary Figure 8C

Cortical neurons

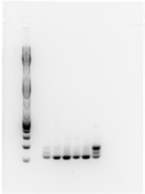

White matter glial cells

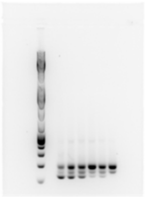

Spinal motor neurons

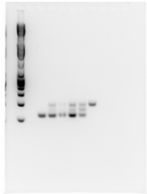

Supplementary Figure 8D

Cortical neurons

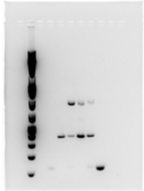

White matter glial cells

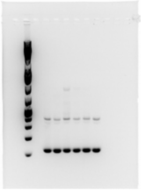

Spinal motor neurons

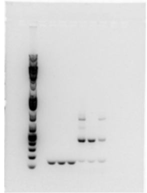

Supplementary Figure 10. Uncropped gel images of Figures 5, 6C, and Supplementary Figure 8.
